# The neglected role of micronutrients in predicting soil microbial structure

**DOI:** 10.1038/s41522-022-00363-3

**Published:** 2022-12-27

**Authors:** Ziheng Peng, Chunling Liang, Min Gao, Yu Qiu, Yanjing Pan, Hang Gao, Yu Liu, Xiaomeng Li, Gehong Wei, Shuo Jiao

**Affiliations:** State Key Laboratory of Crop Stress Biology in Arid Areas, Shaanxi Key Laboratory of Agricultural and Environmental Microbiology, College of Life Sciences, Northwest A&F University, Yangling, Shaanxi 712100 P. R. China

**Keywords:** Soil microbiology, Microbiome, Microbial ecology

## Abstract

Predicting the distribution patterns of soil microbial communities requires consideration of more environmental drivers. The effects of soil micronutrients on composition of microbial communities are largely unknown despite micronutrients closely relating to soil fertility and plant communities. Here we used data from 228 agricultural fields to identify the importance of micronutrients (iron, zinc, copper and manganese) in shaping structure of soil microbial communities (bacteria, fungi and protist) along latitudinal gradient over 3400 km, across diverse edaphic conditions and climatic gradients. We found that micronutrients explained more variations in the structure of microbial communities than macronutrients in maize soils. Moreover, micronutrients, particularly iron and copper, explained a unique percentage of the variation in structure of microbial communities in maize soils even after controlling for climate, soil physicochemical properties and macronutrients, but these effects were stronger for fungi and protist than for bacteria. The ability of micronutrients to predict the structure of soil microbial communities declined greatly in paddy soils. Machine learning approach showed that the addition of micronutrients substantially increased the predictive power by 9–17% in predicting the structure of soil microbial communities with up to 69–78% accuracy. These results highlighted the considerable contributions of soil micronutrients to microbial community structure, and advocated that soil micronutrients should be considered when predicting the structure of microbial communities in a changing world.

## Introduction

One of the urgent transitions in microbial ecology is progressing from the descriptive records of patterns in microbial composition towards a predictive understanding of the shifts in microbial communities in response to environmental changes^1,2^. The composition of these communities is sensitive to surrounding environmental changes, and such shifts would affect their functioning^3,4^. Understanding how the composition of soil communities respond to environment changes is critical for predicting future changes in ecosystem functioning. Unfortunately, the enormous diversity of soil organisms and the complexity of environmental properties create obstacles for our understanding of the underlying dynamics in microbial communities^5^. Over the past decades, a growing number of survey and experimental studies have focused on the effects of climate, plant, soil properties on soil microbial communities across continental and global scale^6–10^. However, a large fraction of variation in soil microbial community’s composition remains unexplained. Unraveling the complexities of the relationship between soil microbial communities and environment requires consideration of more environmental drivers.

In soils, most research has focused on the role of soil pH and macronutrients (e.g., nitrogen, phosphorus and potassium) in explaining the structure of soil microbial communities^11–14^. In contrast, soil metal micronutrients (e.g., Fe, Cu, Mn, Zn) supporting essential biological functions remain poorly investigated in terms of their effects on the structure of soil microbes. Although micronutrients concentrations are as low as milligrams per kilogram or even lower, they are indispensable to plants and play a vital role in cell growth and redox homeostasis^15,16^. They engage in the formation of enzyme-substrate complexes and act as enzyme cofactors in common biochemical pathways, such as the synthesis of protein and biomolecules^17,18^. This points to a potentially significant role of micronutrients in affecting microbial communities’ structure, which had been overlooked in the past. However, comprehensively examining the role of micronutrients in explaining the structure of microbial communities is challenging, not only because the concentrations of many micronutrients have not been systematically investigated, but also because the availability of micronutrients are affected by climate and soil physicochemical properties such as aridity, temperature, pH, organic matter and soil cation exchange capacity^16^. In other words, we have not determined whether micronutrients itself directly shape community structure, or whether other environmental factors that co-vary with soil micronutrients might be indirectly linked with the change in community structure via micronutrients^15^. Therefore, unraveling the unique fraction of the effects of soil micronutrients on microbial communities’ structure will advance our understanding of the complex relationships between microbial communities and the environment.

Different groups of soil organisms respond differently to environmental factors. Climatic variables are recently reported to influence the diversity and composition of fungal communities on global scale^10^. The diversity patterns in fungi are found to be decoupled from bacterial patterns in forest ecosystems, with temperature and primary productivity as the major factors in predicting fungal communities but soil properties such as pH and N:P ratio for bacterial communities^19^. Moreover, the largest proportion of the variance in soil bacterial communities reported in recent study is explained by differences in soil chemistry, particularly soil pH, which is confirmed robust across different spatial scales and land-use types^11,12^. Most bacterial taxa exhibit relatively narrow growth tolerances in soil pH perhaps due to the evolutionary history and life history traits^12^. Protists, which are dominated by consumers, are primarily affected by precipitation^20^. In addition, protistan communities are also associated with bacterial and fungal communities, potentially driven by interactions between them^21,22^. Identifying the relative importance of micronutrients to different groups of soil organisms (such as protists, bacteria and fungi) is crucial to advancing our understanding of soil communities and associated ecosystem functions.

Here, we evaluated the relative contribution of soil micronutrients (total and available iron, zinc, copper, manganese) to the composition (relative abundance of phylotypes) of soil bacterial, fungal and protistan communities across latitudinal gradient over 3400 km. The importance of micronutrients was estimated after accounting for climate, soil properties and macronutrients. We used data from 114 parallel agricultural fields (maize and rice) in eastern China, covering diverse climatic and edaphic gradients. This data contained a set of 30 environmental variables-climate (annual mean temperature and precipitation), soil properties (soil pH, cation exchange capacity, sand, silt, clay, dissolved and total organic matter), macronutrients (total and available nitrogen, phosphorus, potassium and sulfur, nitrate and ammonia nitrogen, C/N, C/P and N/P) and micronutrients (total and available iron, zinc, copper, manganese). These variables were major predictors of soil organisms reported in previous study^9,10,20^. Microbial information on the structure of bacterial, fungal and protistan communities were assessed by 16 S rRNA, ITS and 18 S rRNA gene, respectively. We first tested whether micronutrients could explain a unique portion of the variation in the composition of soil bacterial, fungal and protistan communities after controlling for climate, soil physicochemical properties and macronutrients. Then, we identified the microbial taxa that were driven by micronutrients. Finally, we used machine learning approach to examine whether environmental factors provide markedly better prediction of microbial communities after micronutrients were added.

## Results

### The contribution of micronutrients on microbial communities

**Fig. 1:**
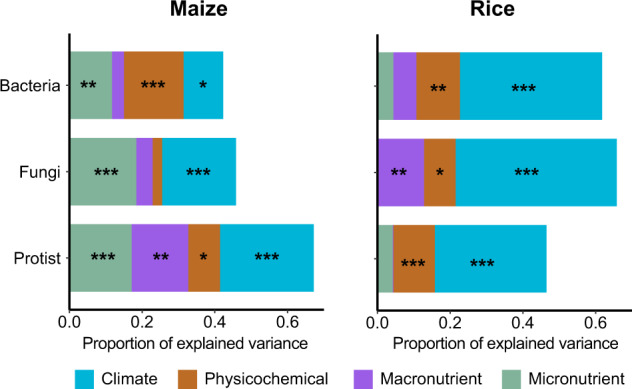
Explained variance of bacterial, fungal and protistan communities in maize and paddy soils by environmental parameters using partial mantel test analysis. Input matrices with variables: climate (mean annual temperature, mean annual precipitation), soil physicochemical properties (soil pH, CEC, sand, silt, clay, dissolved and total organic matter), macronutrients (total and available nitrogen, phosphorus, potassium and sulfur, nitrate and ammonia nitrogen) and micronutrients (total and available iron, zinc, copper, manganese). The significance of statistical test was conducted by partial mantel test analysis based on pearson correlation. Similar results were obtained using multiple regression on matrices (Supplementary Fig. 1). ***p < 0.001; **p < 0.01; *p < 0.05.

We first used partial mantel analysis to quantify the relative contribution of climate, soil physiochemical properties, macronutrients and micronutrients on the structure of bacterial, fungal, and protistan communities. This approach allowed us to estimate the unique contribution of micronutrients on the structure of soil communities by controlling other predictors. Most microbial communities showed strong responses to climate and soil physiochemical properties, explaining 27%, 23%, 34% variance of bacteria, fungi and protists in maize soils, and 51%, 53%, 42% variance in paddy soils, respectively (Fig. 1 and Supplementary Table 1). Most importantly, we found that micronutrients predicted a unique part of the variation in the structure of soil bacterial, fungal and protistan communities in maize soils, accounting for 11%, 18%, and 17% of the variation, respectively (Fig. 1). However, micronutrients had little contribution to predicting the structure of microbial communities in paddy soils, suggesting that land-use change would influence the effects of micronutrients on microbial communities’ composition. Macronutrients, with only significant influence on protist in maize soils and fungi in paddy soils, had a lower ability to predict the structure of microbial communities than micronutrients (Fig. 1). This suggested that micronutrients were better predictor of soil microbial structure than macronutrients. Consistent results were observed when we used multiple regression on matrices (Supplementary Fig. 2 and Supplementary Table 1).

**Fig. 2:**
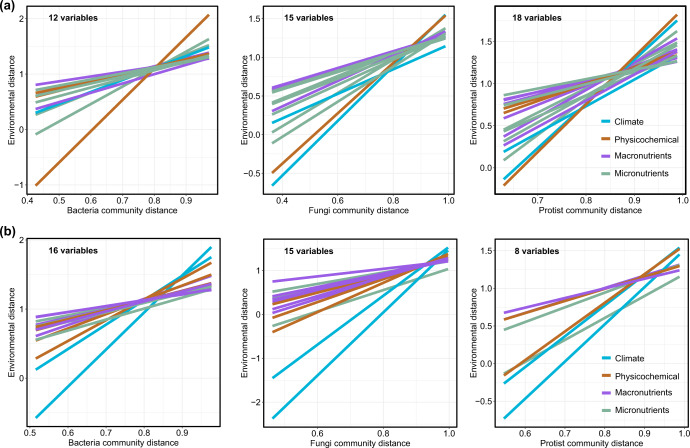
Relationships between soil bacterial, fungal, protistan communities and environmental distance in maize and paddy soils. **a** maize soils, (**b**), paddy soils. Differences among environment and communities were estimated using mantel test base on Bray–Curtis distances. Lines represent fitted linear regressions between environmental distance and associated bacterial, fungal or protistan communities. Only environmental factors with significant relationship with the communities are shown in figure (the number in the upper left). A full version of this figure showing each environmental factor can be found in Supplementary Figs. 2–7 and Supplementary Tables 2–4.

The relationship between the distances in each environmental predictor and microbial communities was then assessed to identity the major environmental factors in structuring bacterial, fungal and protistan communities. The concentrations of several different micronutrients were found to be correlated with the structure of microbial communities, together with climate and soil properties such as mean annual temperature and precipitation, and soil pH (Fig. 2 and Supplementary Figs. 3–8). Specially, bacterial communities were associated with available iron (Pearson: *R* = 0.258, *P* < 0.001), total copper (Pearson: *R* = 0.101, *P* < 0.05), total zinc (Pearson: *R* = 0.091, *P* < 0.05), total manganese (Pearson: *R* = 0.120, *P* < 0.01), and available manganese (Pearson: *R* = 0.197, *P* < 0.001) in maize soils. Fungal communities were associated with total iron (Pearson: *R* = 0.159, *P* < 0.01), available iron (Pearson: *R* = 0.168, *P* < 0.001), total copper (Pearson: *R* = 0.105, *P* < 0.05), available copper (Pearson: *R* = 0.086, *P* < 0.05), total zinc (Pearson: *R* = 0.090, *P* < 0.05), total manganese (Pearson: *R* = 0.120, *P* < 0.01), and available manganese (Pearson: *R* = 0.118, *P* < 0.01) in maize soils. Protistan communities were associated with total iron (Pearson: *R* = 0.141, *P* < 0.01), available iron (Pearson: *R* = 0.269, *P* < 0.001), total copper (Pearson: *R* = 0.089, *P* < 0.05), available copper (Pearson: *R* = 0.104, *P* < 0.05), total manganese (Pearson: *R* = 0.190, *P* < 0.01), and available manganese (Pearson: *R* = 0.189, *P* < 0.001) in maize soils. These results suggested the considerable role of these micronutrients in explaining microbial structure in maize soils. Moreover, available iron showed the strongest relationship with microbial communities among all micronutrients in maize soils (Supplementary Table 2), suggesting the potential importance of iron to influence microbial community structure. For paddy soils, available zinc was correlated with the structure of bacterial, fungal and protistan communities (Supplementary Table 3).

We further used structural equation modeling to disentangle the direct effect of micronutrients and indirect role of other environmental predictors via micronutrients on the changes in microbial communities. To rigorously determine the unique importance of micronutrients, SEM model was constructed representing the effects of different environmental variables in three steps (Supplementary Fig. 9). Soil physicochemical and climatic properties were reported as main predictors for the structure of bacterial, fungal and protistan communities, and thus their effects were tested first as SEM model I. In addition, the influence of macronutrients (e.g., N, P, K, S) on microbial communities have also been widely studied and considered to be important predictors, and their effects were therefore added as SEM model II. Finally, micronutrients were added into SEM model III. To match the SEM model, we used non-metric multidimensional scaling (NMDS) to transform ASVs abundance data into low-dimensional space and kept the first two axes for bacterial, fungal and protistan communities, respectively. The micronutrients were correlated with the NMDS axis scores of bacterial, fungal, and protistan communities (Supplementary Fig. 10).

**Fig. 3:**
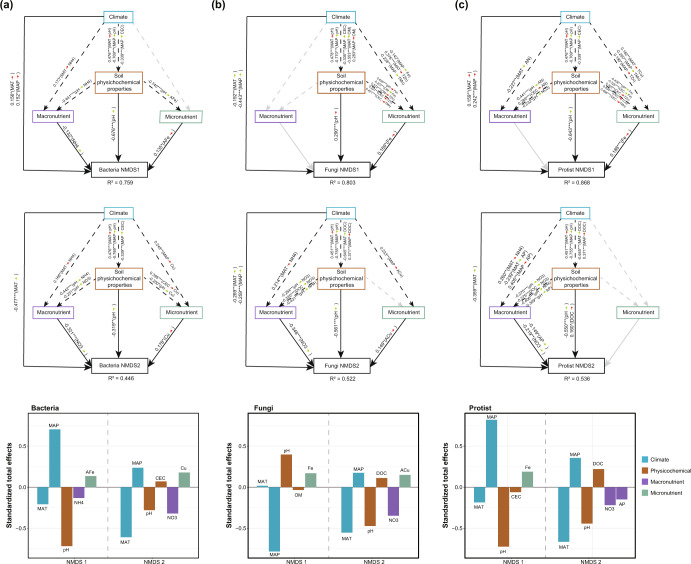
Structural equation models identifying the direct (full lines) and indirect (dotted lines) influence of different predictors on the structure of bacteria, fungi and protistan communities in maize soils. **a** bacteria, (**b**) fungi, (**c**), protist. Black lines indicate significant and numbers adjacent to lines were indicative of the effect size of the relationship. We grouped the climatic and edaphic properties into the same box in the model for graphical simplicity, which did not represent latent variables. R^2^ denotes the proportion of variance explained. Red arrows represented positive paths, and green arrows represented negative paths. Significance levels were as follows: *p < 0.05, **p < 0.01, and ***p < 0.001. The total standardized effects on SEM on the structure of microbial communities were calculated as sum of the direct and indirect effects. Information about our a priori model was provided in Supplementary Fig. 8. TZn total zinc, AZn available zinc, TFe total iron, AFe available iron, TCu total copper, ACu available copper, TMn total manganese, AMn available manganese, CEC cation exchange capacity.

Our final SEM analyses showed that micronutrients had direct effects on the first and second axis of bacteria (iron and copper), the first and second axis of fungi (iron and copper), and the first axis of protists communities (iron) in maize soils after accounting for other predicting factors simultaneously, with the sole exception of protistan second axis (Fig. 3). These results indicated that micronutrients explained a unique proportion of the variation in the structure of microbial communities, although climate and soil physicochemical properties showed the largest total effects on microbial structure. Moreover, climate and soil physicochemical properties also indirectly affected the microbial communities through their effects on the composition and concentration of micronutrients, suggesting the mediating role of micronutrients between climate, soil and microbial structure (Fig. 3). For example, indirect effects of soil pH on microbial structure were largely driven by micronutrients such as iron (negative and positive effect of pH in bacterial and fungal axis one, respectively). Climate such as MAP indirectly drove microbial communities’ structure through its effect on soil micronutrient concentrations (positive effects on copper in bacterial and fungal second axis). When SEM analyses were repeated using data from paddy soils (Supplementary Fig. 11), we found that a unique part of the variance of microbial communities was still predicted by micronutrients. However, micronutrients had a lower ability to predict the structure of microbial communities in paddy soils than that in maize soils (two of six axis scores), with iron for fungal axis one and manganese for protistan second axis.

### Microbial phylotypes influenced by micronutrients

**Fig. 4:**
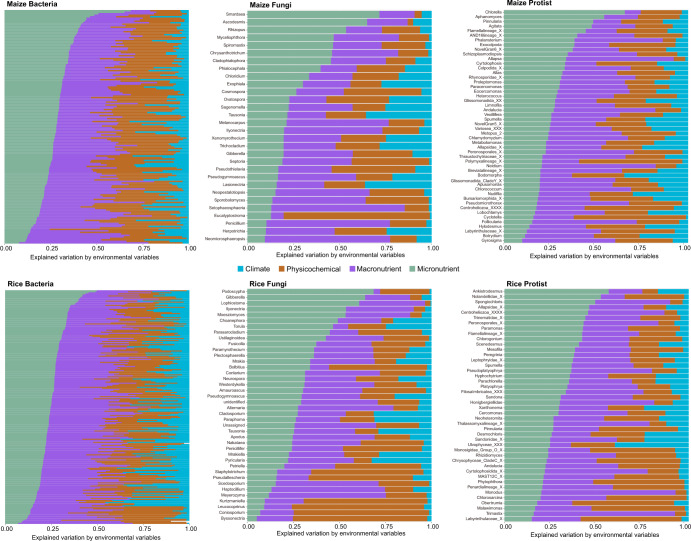
Environmental variables explaining the structure of bacterial, fungal and protistan communities. Contribution of climate, soil physicochemical properties, macronutrients and micronutrients to the variation explained by the complete random forest model for each microbial genus where out-of-bag *R*^*2*^ > 0.

Random forest analyses were conducted to identify microbial phylotypes at ASV (Supplementary Figs. 12 and 13) and genera (Fig. 4 and Supplementary Figs. 14–20) levels that were primarily affected by micronutrients. For example, genus Gibberella as plant pathogens was found to be an indicator of changes in micronutrients and the relative abundance decreased with the concentrations of available iron, zinc, copper and manganese (Supplementary Fig. 21). Of the environmental factors, micronutrients accounted for more than 25% of variability over half of the phylotypes (Fig. 4). Across bacterial, fungal and protistan communities, the random forest models were sensitive to multiple micronutrients abundance, such as available manganese for bacteria and protist in maize soils and available copper for fungi in paddy soils (Supplementary Fig. 22).

### The importance of micronutrients by machine learning

**Fig. 5:**
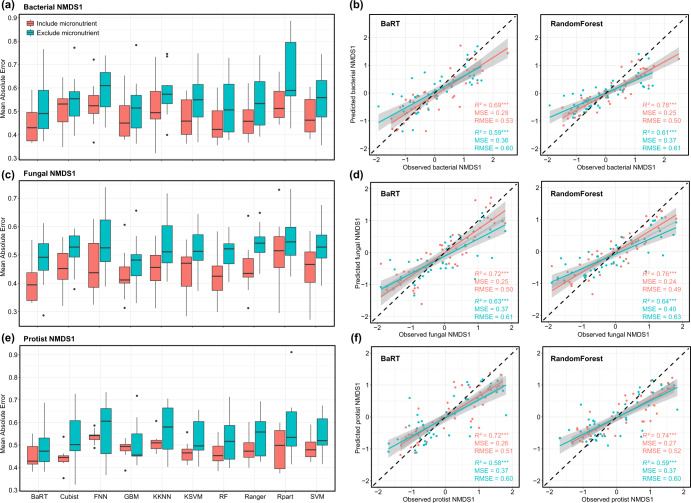
Comparison of prediction errors of different machine learning algorithms for the structure of bacterial, fungal and protistan communities. Machine learning algorithms include Bagged Regression Tree (BaRT), Cubist, Fast Nearest Neighbor (FNN), Gradient Boosting Machines (GBM), Weighted k-Nearest Neighbor (KKNN), Kernel Support Vector Machine (KSVM), Random Forest (RF), Ranger, Rpart and Support Vector Machine (SVM), showing bacterial (**a**), fungal (**c**), and protistan communities (**e**). The center line of boxplot represents the median of data. The boxplot bounds the interquartile range (IQR) divided by the median, and whiskers extend to a maximum of 1.5 times the IQR beyond the box. Other observed data points outside the boundary of the whiskers are plotted as outliers, shown a dot. Predicted vs. observed the structure of bacterial (**b**), fungal (**d**) and protistan (**f**) communities of test dataset derived from bagged regression tree (BaRT) and random forest (RF). R^2^, coefficient of determination; MSE, mean squared error; RMSE, root mean squared error. The significance of statistical test was conducted by BaRT and RandomForest regression models. *p < 0.05, **p < 0.01, and ***p < 0.001.

To validate importance of micronutrients, we further used machine learning approach to evaluate whether the inclusion of micronutrients could provide a better, higher accuracy in predicting the structure of microbial communities in agricultural fields (Fig. 5 and Supplementary Fig. 23). Machine learning (ML) algorithms provided powerful tools for explaining multivariate, nonlinear, and non-monotonic relationships^23,24^. Ten classic ML algorithms - Bagged Regression Tree (BaRT), Cubist, Fast Nearest Neighbor (FNN), Gradient Boosting Machines (GBM), Weighted k-Nearest Neighbor (KKNN), Kernel Support Vector Machine (KSVM), Random Forest (RF), Ranger, Rpart and Support Vector Machine (SVM) were employed to assess the prediction performance of microbial communities’ models. As expected, we found that the models including micronutrients had a lower mean absolute error (median of 0.483), mean squared error (median of 0.415) and root mean squared error (median of 0.633) than those obtained from the model excluding micronutrients in predicting the structure of bacterial, fungal and protistan communities (Fig. 5 and Supplementary Tables 4–6), suggesting that the addition of micronutrients substantially increased the predictive power of environmental variables. Moreover, ensemble machine learning methods such as BaRT and RF showed a lower mean absolute error (BaRT: 0.445; RF: 0.446) and root mean squared error (BaRT: 0.593; RF: 0.584), yielding better prediction accuracy compared to other methods. The observed vs. predicted the structure of bacterial, fungal and protistan communities in test dataset matched well for RF and BaRT with values aligned close to the 1:1 line in models including micronutrients than those values in models excluding micronutrients (Fig. 5).

## Discussion

Together, our results provided empirical evidence that micronutrients could leave a considerable effect on the structure of soil bacterial, fungal and protistan communities. Our work highlighted that micronutrients explained a unique and significant portion in variation in soil microbial communities beyond what could be predicted from climate, soil physicochemical properties or macronutrients. These results emphasized the fact that micronutrients could be used to gain a better and more accurate understanding and predicting in the composition of soil communities, which was helpful to understanding mechanisms of microbial communities to the surrounding environment, including micronutrients gain and loss, and fertilization strategies. For example, our results suggested that concentrations of iron and copper might directly affect bacterial, fungal and protistan communities independent of the climate, soil physicochemical properties and macronutrients in maize soils. This result was consistent with previous study demonstrating that iron and its complexes affected microbial community structure, with increasing bacterial interactions and the number of mutually beneficial taxa in soils^25^. Moreover, experimental studies dedicating to the study of organic fertilizers mainly focused on the effects of organic carbon and macronutrients (N, P and K) on microbial communities while ignoring micronutrients^26,27^. However, the applications of organic fertilizers were also linked with soil micronutrients status in soils^28^. A recent global grassland study showed that nitrogen and phosphorus fertilization favored fungal pathogens^29^, in contrast to our study that Gibberella decreased with abundance of micronutrients. These results suggest that the effects of organic fertilizers on microbial communities are complex, and the combined effects of both macro- and micronutrients should be considered.

In the present study, the structure of fungal and protistan communities showed stronger relationships with micronutrients than the bacterial communities, which might attribute to physiological and ecological differences among these groups. Bacterial communities composition was reported to be primarily determined by soil physicochemical properties such as soil pH^11^, consistent with our results. The effects of other environmental variables on bacterial communities were far weaker than those of soil properties. In addition, the concentration of available iron was closely linked with bacterial communities, which might be associated with ubiquitous iron-reducing and oxidizing bacteria that determined bioavailability of iron^30^. Fungal communities are key decomposers of plant necromass and symbionts of plant growth involving soil micronutrient cycles, and tend to be largely driven by plant communities and the climate associated with plant distribution^10,31^. Arbuscular mycorrhizal fungi increases ferric chelate reductase activity as well as Fe, Zn, S and P in plant under Fe-deficiency, which is related to the bioavailability of Fe at rhizosphere zone^32^. Fungi are more effective than acidophilic autotrophic and heterotrophic bacteria for bioremediation of heavy metals (Zn and Cu) in sediments^33^. Unlike bacteria and fungi, the composition of protists is dominated by consumers and is found to be jointly structured by climate, soil physicochemical properties, macronutrients, micronutrients and its prey^20,34^.

As reported, palaeoclimate, current climate, soil development, soil chemistry, topography, and vegetation account for a large variation in the structure of microbial communities^9,10,35–37^. However, micronutrients were demonstrated to explain a unique and significant part of variation in our study. In addition, climate and soil properties could also indirectly alter microbial communities’ structure by regulating micronutrients, suggesting that climate and soil properties cannot account solely (via direct effects) for soil microbial communities. For example, the SEM models showed that, besides the direct effect, soil properties had an indirect effect on fungal and protistan communities through their negative effect on soil iron concentrations. Linkages of micronutrients to microbial communities advance our understanding of the pathways by which climatic and soil properties affect microbial communities and how microbial communities respond to ongoing global changes. Nevertheless, there was still a large proportion of unexplained variations in microbial communities structure, which might be attributed to stochastic processes such as homogenizing dispersal and ecological drift and unconsidered factors such as crop genotypes^38^.

Moreover, micronutrients had a lower ability to predict the structure of microbial communities in paddy soils than in maize soils. This suggested that different agricultural practices and crop types had distinct effects on the unique contribution of micronutrients as a predictor of microbial structure. Compared to maize soils, paddy fields are subject to more agricultural disturbances, such as experiencing more human-driven physical practices and suffering larger fluctuations in soil moisture. Soil moisture is one of major drivers on microbial communities structure^39,40^, and drought-rewetting events might attenuate the direct contribution of micronutrients on soil microbial communities by directionally promoting moisture-insensitive taxa^41,42^.

Overall, our work provides fundamental insights into the complexities of relationships between environment and soil microbial communities, and quantifies the relative contribution of micronutrients in predicting the structure of bacterial, fungal and protistan communities. We show that soil micronutrients explain a unique proportion of the variation in microbial communities’ structure even after controlling for climate, soil physicochemical properties and macronutrients. Our results from machine learning indicate that the inclusion of micronutrients in the model increases our ability to predict the structure of soil microbial communities. Thus, given the important role of micronutrients in predicting microbial communities, information on micronutrients is essential to fully unravel the complexity relationships between environment and microbial communities and the impact of global changes on belowground communities.

## Methods

### Data collection and soil analysis

We used data from agricultural fields under long-term cultivation with maize and paddy soils across eastern China^43,44^. The dataset included 114 paired sites, amounting to 114 maize and 114 paddy soil samples along a wide latitudinal gradient over 3400 km, across diverse edaphic conditions and climate gradient. Collected paddy soils are around maize soils (within 5 km) in each paired site (Supplementary Fig. 1). The soil samples were collected during the growing season (July–September 2017). In each site, a composite soil sample were composed of 15 soil cores taken at a depth of 0–15 cm from three plots (each 100 m^2^) and was separated into two portions. One portion was air-dried and used for soil physicochemical properties, macronutrients and micronutrients analyses, and the other was stored at − 80 °C until DNA extraction.

For all soil samples, we measured soil physicochemical properties (soil pH, CEC, sand, silt, clay, dissolved and total organic matter), macronutrients (total and available nitrogen, phosphorus, potassium and sulfur, nitrate and ammonia nitrogen, C/N, C/P and N/P) and micronutrients (total and available iron, zinc, copper, manganese). Soil properties were determined using standardized protocols^45,46^. Briefly, soil texture was determined using a simple sifting and sedimentation-based approach^47^, and the pH of the soil was assessed in a 1:5 suspension (soil to distilled water). Organic matter was determined colorimetrically following oxidation with a combination of potassium dichromate and sulfuric acid^48^. Dissolved organic carbon (DOC) was measured by high-temperature combustion, the high temperature (110 °C) conversion of inorganic carbon to dissolved CO_2_, and purging this from the sample, the remaining (organic) carbon is then oxidized at a high temperature to CO_2_ which can be detected by the instrument’s nondispersive infrared (NDIR) sensor^49^. Cation exchange capacity (CEC) of the soil was measured by a modified ammonium-acetate compulsory displacement^50^. Total nitrogen (TN) was determined using a Flash 2000 NC Analyzers (Thermo Scientific, MA, USA). Ammonium (NH_4_^+^) and nitrate (NO_3_^−^) concentrations in extracts were determined colorimetrically by automated segmented flow analysis (AAIII; Bran and Luebbe, Germany). Determination of total phosphorus (TP) and total potassium (TK) were used potassium dichromate-sulfuric acid digestion and ammonium acetate-flame photometry method, respectively^51^. Available phosphorus (AP) was extracted by 0.5 M NaHCO_3_ and determined using the molybdenum blue method. Available potassium (AK) was determined in 1 M ammonium acetate extracts by flame photometry (FP640, INASA, China). After digestion with HNO_3_/H_2_O_2_ mixture and extraction with DTPA^52^, we measured total and available metal concentrations using inductively coupled plasma atomic emission spectroscopy (Iris Intrepid II XDL; Thermo Fisher Scientific). Because the DTPA method is widely utilized in neutral to basic soils, as is the case for most of the soils investigated, and the DTPA extraction for copper, iron, manganese, and zinc is standardized, so we used it as an indication of the accessible pool of metals in soils and limited our analysis to them. We obtained climatic data including mean annual temperature (MAT) and mean annual precipitation (MAP) based on the sampling sites from the Worldclim database (www.worldclim.org).

### Molecular analysis

Soil bacterial, fungal and protistan communities were analyzed using high-throughput sequencing. Total genomic DNA was extracted from soil samples using a FastDNA SPIN Kit for Soil (MP Biochemicals, Solon, OH, USA). Microbial communities were profiled by targeting the V4-V5 region of 16 S rRNA gene for bacteria, a region of the internal transcribed spacer 1 gene for fungi, and the V4 region of 18 S rRNA in protists. Corresponding polymerase chain reaction assays were performed with the 515 F/907 R (~450 bp)^53^, ITS5-1737F/ITS2-2043R (~300 bp)^54^ and TAReuk454FWD1/TAReukREV3 (~380 bp)^55^ primer pairs, respectively^44^. PCR amplification was performed in a 50 μl volume: 25 μl 2x Premix Taq (Takara Biotechnology, Dalian Co. Ltd., China), 1 μl each primer (10 μM) and 3 μl DNA (20 ng/μl) template. The PCR thermal cycling conditions were performed by thermocycling: 5 min at 94 °C for initialization, followed by 30 cycles of 30 s denaturation at 94 °C, 30 s annealing at 52 °C, 30 s extension at 72 °C, and 10 min final elongation at 72 °C. The length and concentration of the PCR product were detected by 1% agarose gel electrophoresis. Sequencing libraries were generated using NEBNext® Ultra™ II DNA Library Prep Kit for Illumina® (New England Biolabs, MA, USA) following manufacturer’s recommendations and index codes were added. Sequencing was performed on the Illumina HiSeq2500 platform (Illumina Inc., San Diego, CA, USA). Bioinformatics processing, including filtering, dereplication, sample inference, chimera identification, and merging of paired-end reads, was performed using the Divisive Amplicon Denoising Algorithm 2 (DADA2)^56^, a model-based approach for correcting Illumina amplicon errors without constructing OTUs. Compared to other methods, DADA2 identified more real variants and output fewer spurious sequences. Taxonomical annotation of the representative sequences of amplicon sequence variants (ASVs) was performed with a naïve Bayesian classifier using the Silva v. 138 for bacteria^57^, UNITE + INSD v. 8.3 for fungi^58^, and Protist Ribosomal Reference database v. 4.13.0 for protist^59^. Bacteria and protists were defined as all prokaryotic and eukaryotic taxa, except Archaea, chloroplasts, mitochondria in 16 S gene and Rhodophyta, Streptophyta, Metazoa and fungi in 18 S gene for subsequent analyses^20,60^.

### Correlation and regression analyses

We first examined the relationship between environmental variables and the bacterial, fungal and protistan communities through partial Mantel test and multiple regression on distance matrices using the “ecodist” package in R^61,62^, as shown Supplementary Table 1. To quantify dissimilarity in bacterial, fungal and protistan communities across sampling sites, dissimilarity matrices based on Bray–Curtis dissimilarities of ASVs were calculated. Here we used four environmental explanatory matrices: climate variables (annual mean temperature and precipitation), (2) soil physicochemical properties (soil pH, cation exchange capacity, sand, silt, clay, dissolved and total organic matter), (3) macronutrients (total and available nitrogen, phosphorus, potassium and sulfur, nitrate and ammonia nitrogen) and (4) micronutrients (total and available iron, zinc, copper, manganese). To quantify dissimilarity in climate, soil properties, macronutrients and micronutrients across sampling sites, we calculated Euclidean dissimilarities. Four distance matrices were calculated that represent climate, soil properties, macronutrients and micronutrients. Each environmental matrix was individually associated with the microbial communities based on Pearson’s correlations, leaving three environmental variables as controlling distance matrix in partial Mantel test, as shown in Fig. 1. Multiple regression analysis was performed on the four distance matrices and microbial communities, as shown Supplementary Fig. 2. Mantel test was further used to assess the significance of the association between each environmental predictor of 30 variables and bacterial, fungal and protistan communities, respectively, as shown in (Fig. 2).

### SEM analyses

We used structural equation modeling that synthesized prior knowledge in model to disentangle the direct and indirect role of the complex relationships among climate, soil physicochemical properties, macronutrients, micronutrients and microbial community. Structural equation modeling allowed us to partition causal influences among multiple predictors and separated the direct and indirect effects of model predictors on the structure of microbial community. Variables were log-transformed and standardized prior to improve normality and linearity.

To rigorously evaluate the relative effects of micronutrients on the structure of microbial community, three SEMs representing the effects of different variables were constructed stepwise (Supplementary Fig. 9), as shown in previous study that micronutrients have important role on global biomass production^15^. First, climatic and soil physicochemical properties were incorporated in model and tested, as temperature, precipitation and soil properties were expected to be the important drivers of the composition of microbial communities^11,63^. Second, the effects of macronutrients (such as N, P, and K) on microbial communities had also been extensively studied and were thus incorporated into the model^64,65^. Finally, we tested whether micronutrients explained additional variation in microbial communities due to their important role in life chemistry and ecosystem functioning^16,66^. Specifically, MAT and MAP were firstly used to build the model together with soil physicochemical properties SOM, DOC, CEC, pH and texture. Each of the soil physicochemical properties was added separately to the model including climatic factors. All those that significantly contributed (*p* < 0.05) to explaining additional variation were retained, as following a similar approach in^15,67^. In the following steps, nitrogen, phosphorus, potassium and sulfur were separately added to the previous model and those that had a significant contribution were retained. The same procedure was applied in the next step for micronutrients (iron, zinc, copper, and manganese). When both total and available metal were retained, the metal was grouped into one composite variable by summing the product of total and available metal with their coefficient in the full SEM model. The model was then reconstructed substituting the individual metal with the composite variable. We grouped the different categories of predictors (climate, soil physicochemical properties, macronutrients and micronutrients) in the same box in the model for graphical simplicity, but these boxes did not represent latent variables.

Three metrics were used to quantify the goodness of fit of SEM models: the χ2 test, the Root Mean Square Error of Approximation (RMSEA), and Comparative Fit Index (CFI). Specially, the closer to 1 CFI value, closer to 0 RMSEA values, and higher χ2 and RMSEA P values, the better model perform. With a good model fit, we were able to interpret the path coefficients of the model and their associated P values. A path coefficient was analogous to the partial correlation coefficient, and described the strength and sign of the relationship between two variables. Structural equation models were constructed using the “lavaan” package in R^68^.

### Randomforest modeling

To explore the environmental predictors of microbial phylotypes and genera, we modeled distribution of each phylotype or genus by Random Forest^69^. Many of the predictor variables used in ecological studies were either naturally (e.g., the availability of metal with total metal) or functionally (e.g., soil moisture was calculated as a function of precipitation and evapotranspiration) correlated. While some of these predictors may determine species distribution or abundance other collinear predictors may not. The random subset approach for fitting predictor variables at each node could result in a correlated but less in influential predictor standing in for more highly in influential predictors in the early splits of an individual tree depending upon which predictor is selected in the subset. The overall predictive ability of the forest for each phylotype or genus was calculated as the average proportion of out-of-bag data variance explained by the fitted forest. In model, the accuracy importance of predictor was quantified as the decrease in performance when each predictor was randomly permuted but other predictors are not modified. To show variable importance across all modeled phylotypes or genera, the relative importance of each predictor was calculated as a sum of predictor relative importances of all Random Forests for individual phylotype or genus weighted by Random Forest predictive ability (out-of-bag R^2^)^10,70,71^. Monotonic function of each predictor that delineates where the compositional change points occurred along the gradient of the predictor was obtained.

### Modeling techniques

Machine learning (ML) algorithms were powerful tools for explaining multivariate, nonlinear, and non-monotonic relationships. We further used machine learning approach to test whether environmental factors provide markedly better prediction accuracies of microbial communities when micronutrients were added. Ten classic ML algorithms (i.e., Bagged Regression Tree (BaRT), Cubist, Fast Nearest Neighbor (FNN), Gradient Boosting Machines (GBM), Weighted k-Nearest Neighbor (KKNN), Kernel Support Vector Machine (KSVM), Random Forest (RF), Ranger, Rpart and Support Vector Machine (SVM)) were selected for regression prediction to assess the prediction performance of microbial communities’ models. Our aim was to assess whether environmental factors including micronutrients provided better prediction accuracies than environmental factors excluding micronutrients, and thus environment factors was divided into two group: including micronutrients (climate, soil physicochemical properties, macronutrients and micronutrients) and excluding micronutrients. Five-fold cross-validation was calculated to estimate test errors of the models. For each model, 80% of the dataset was randomly separated into a training set, with the remaining 20% used as a testing set. Three performance parameters were calculated simultaneously to evaluate the machine learning models, including mean absolute error (MAE), mean squared error (MSE), and root mean squared error (RMSE) obtained from the test data set. Lower value represented better the prediction performance of models. Machine learning methods were conducted using the “mlr3” package in R^72^.

### Reporting summary

Further information on research design is available in the Nature Research Reporting Summary linked to this article.

## Supplementary information

Supplementary information

Reporting Summary

Dataset 1

## Data Availability

Environmental data are archived in 10.6084/m9.figshare.19765978.v1. Sequencing data are accessible on GenBank via BioProject accession PRJNA544819.

## Code availability

R code used for data processing, graph creation, and statistical analyses related to this study are available on GitHub at https://github.com/Pong2021/The-neglected-role-of-micronutrients-in-predicting-soil-microbial-structure.
