# Supplementary material for: The neglected role of micronutrients in predicting soil microbial structure
Source: NPJ Biofilms Microbiomes. 2022 Dec 27;8:103. doi: 10.1038/s41522-022-00363-3 (PMC9794713; doi:10.1038/s41522-022-00363-3)
Supplement: Supplementary file 1 — Supplementary information [file 41522_2022_363_MOESM1_ESM.pdf]

Supplementary information

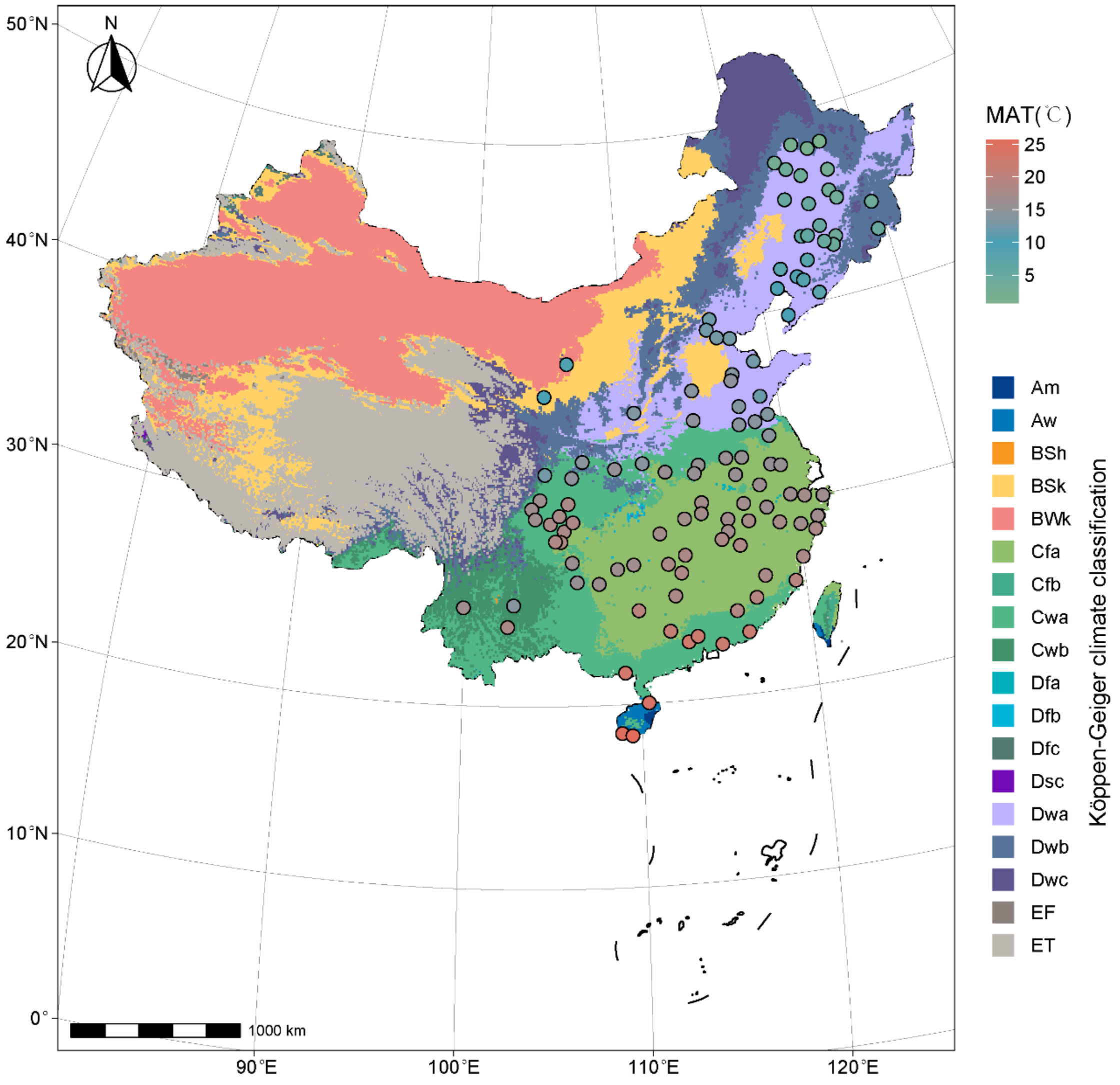

Supplementary Figure 1. The parallel 114 sampling locations of maize and paddy soils across climate regions. The color background represents the Köppen-Geiger climate classification (www.gloh2o.org/koppen).

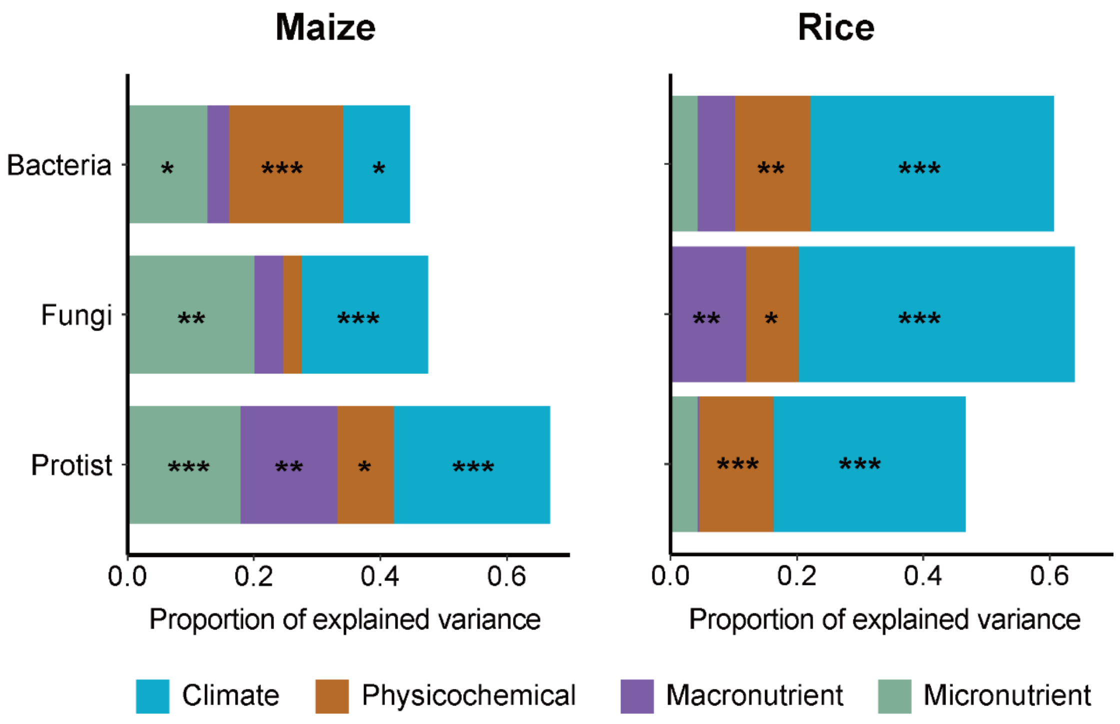

Supplementary Figure 2. Explained variance of bacterial, fungal and protistan communities in maize and paddy soils by environmental parameters using multiple regression on matrices. Input matrices with variables: climate (mean annual temperature, mean annual precipitation), soil physicochemical properties (soil pH, CEC, sand, silt, clay, dissolved and total organic matter), macronutrients (total and available nitrogen, phosphorus, potassium and sulfur, nitrate and ammonia nitrogen, C/N, C/P and N/P) and micronutrients (total and available iron, zinc, copper, manganese). ***, $p < 0.001$; **, $p < 0.01$; *, $p < 0.05$.

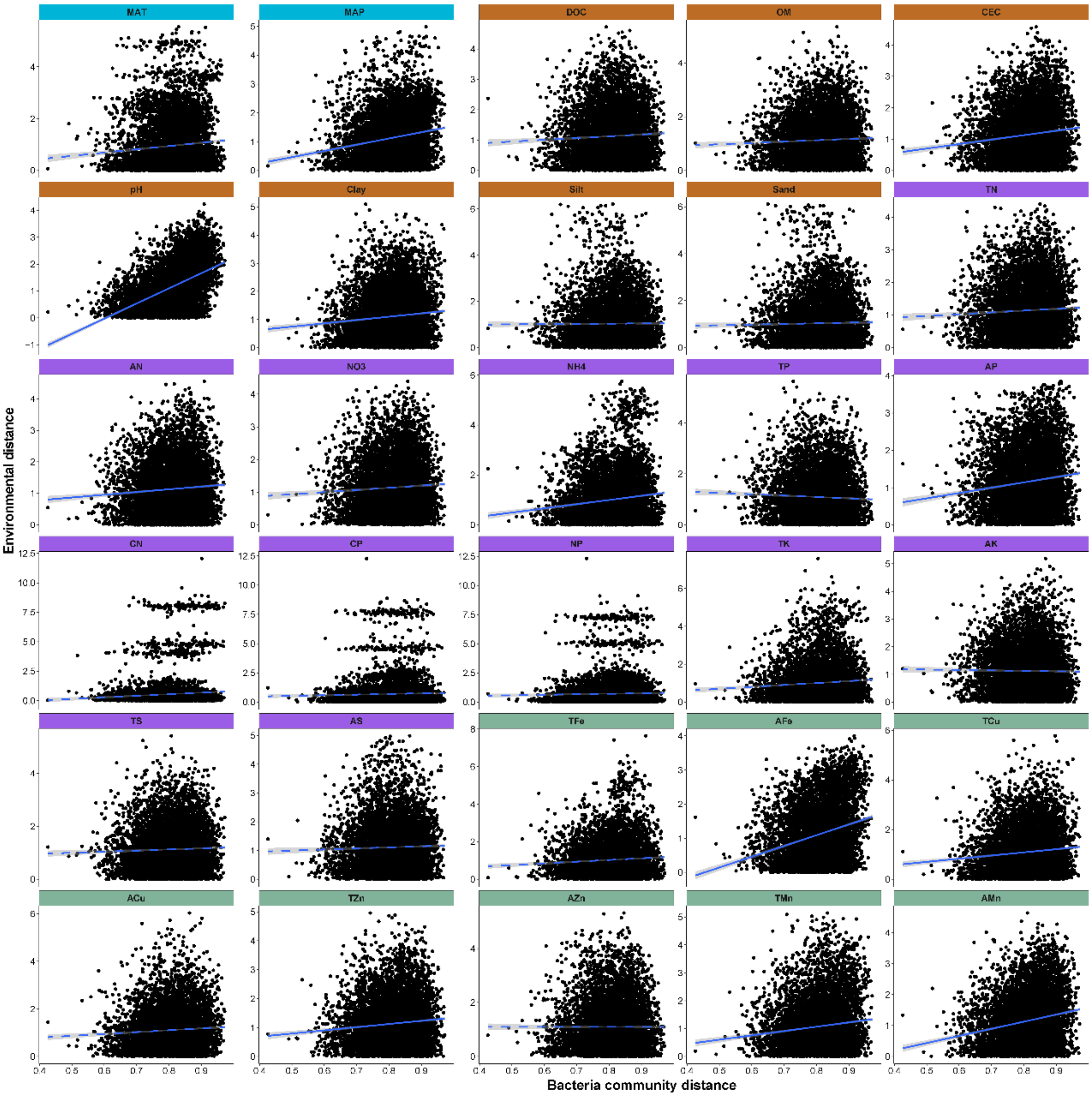

Supplementary Figure 3. Relationships between soil bacterial communities and environmental distance in maize soils. Dashed lines represent non-significant relationships.

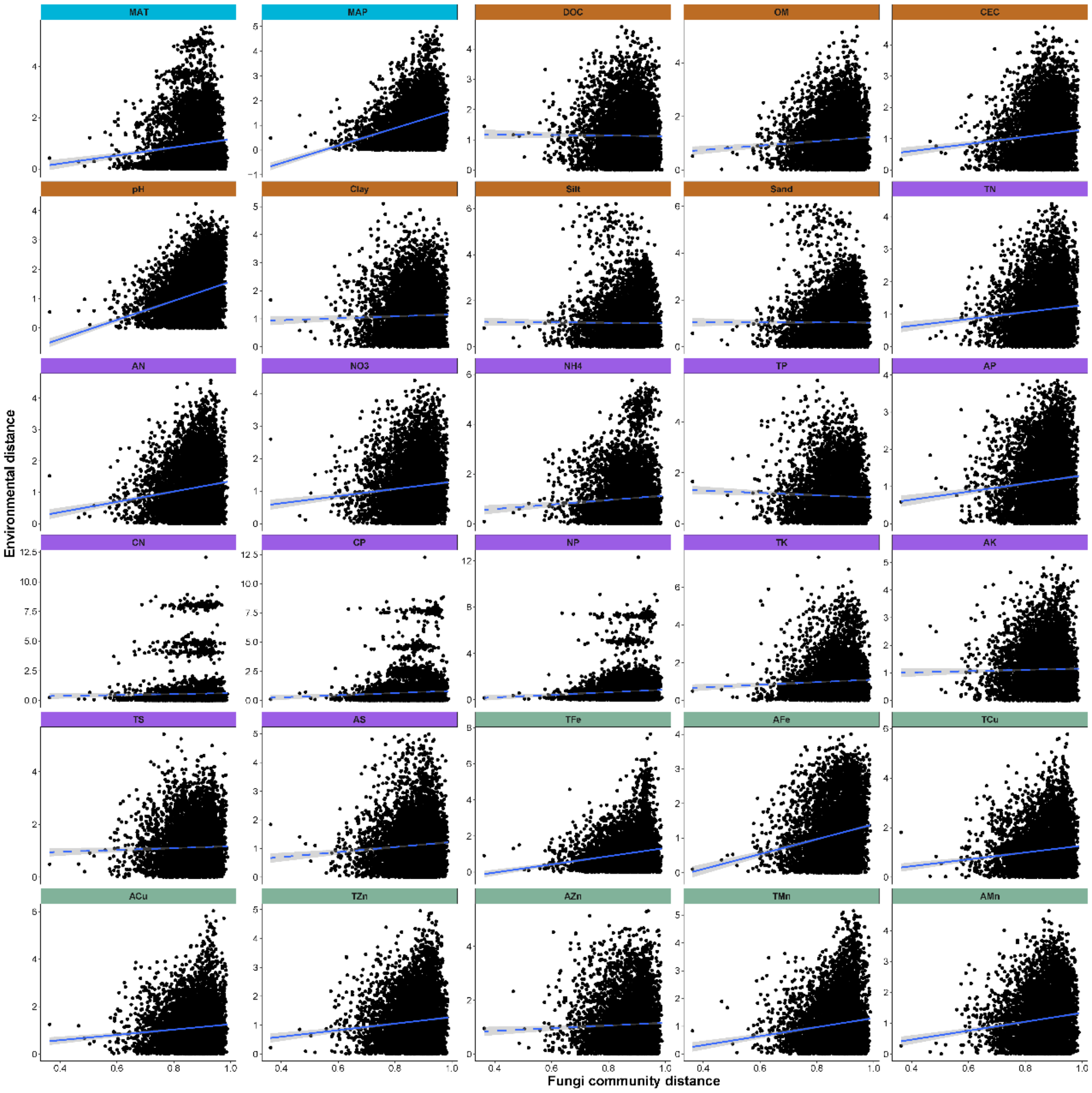

Supplementary Figure 4. Relationships between soil fungal communities and environmental distance in maize soils. Dashed lines represent non-significant relationships.

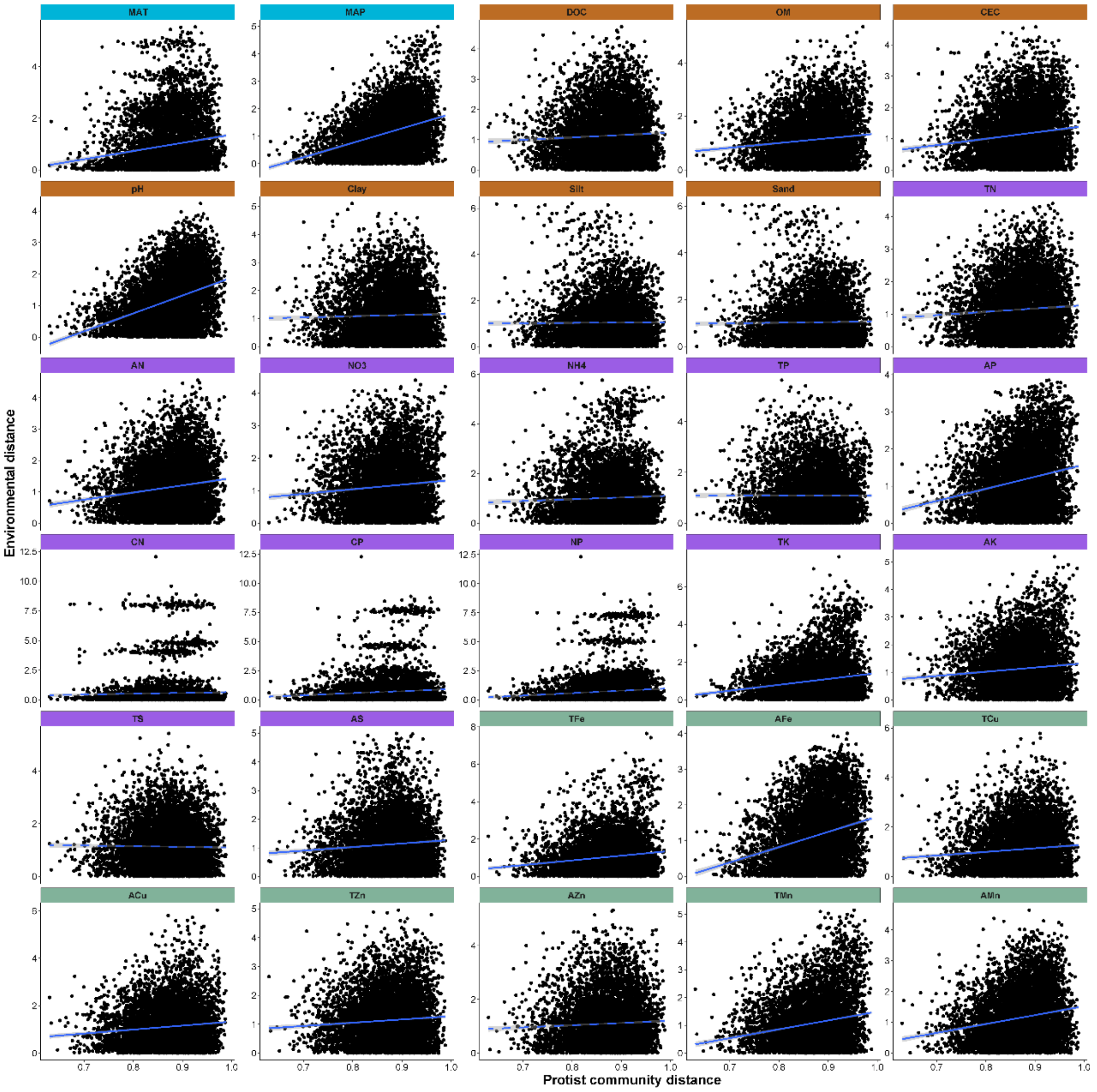

Supplementary Figure 5. Relationships between soil protistan communities and environmental distance in maize soils. Dashed lines represent non-significant relationships.

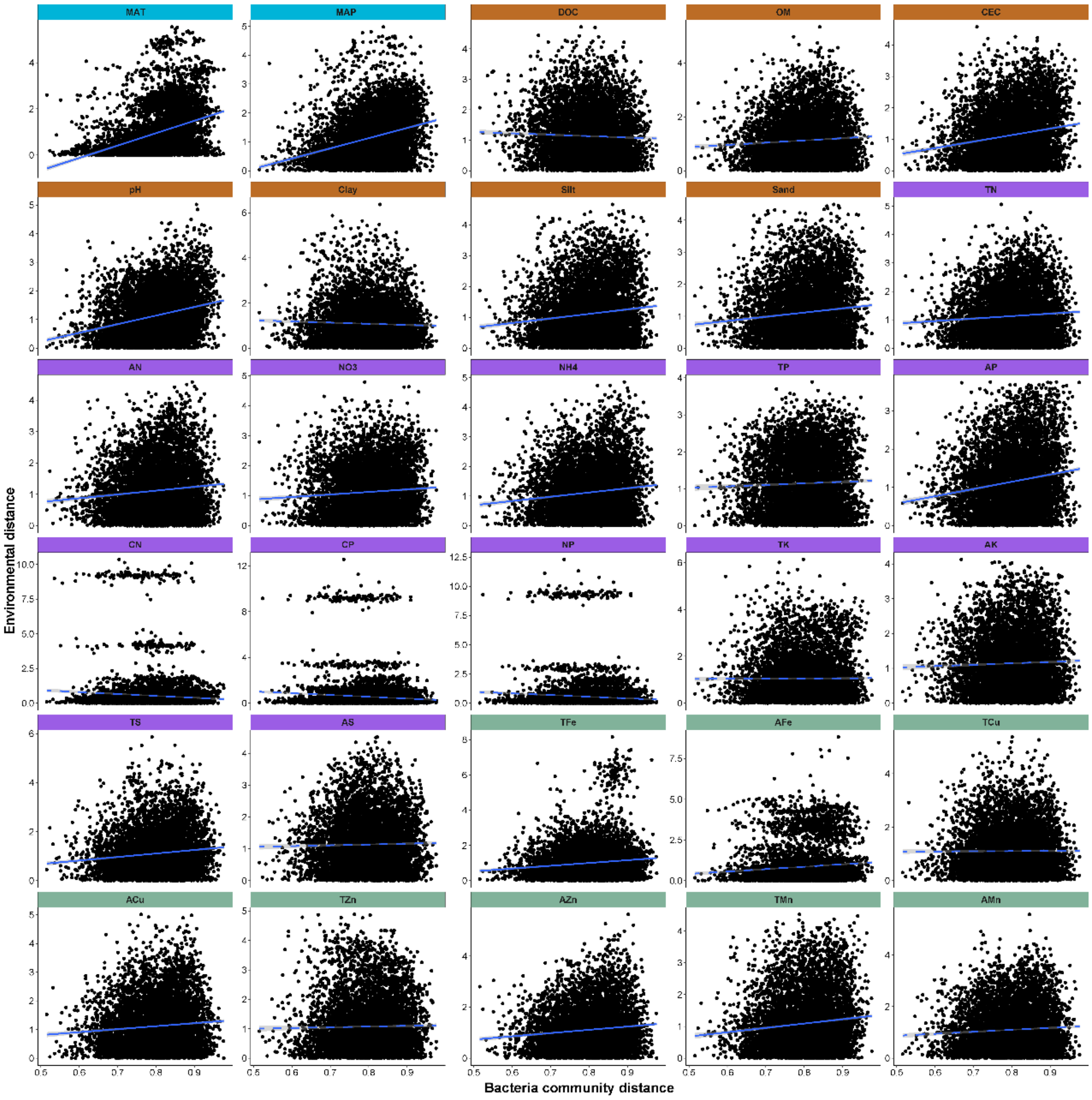

Supplementary Figure 6. Relationships between soil bacterial communities and environmental distance in paddy soils. Dashed lines represent non-significant relationships.

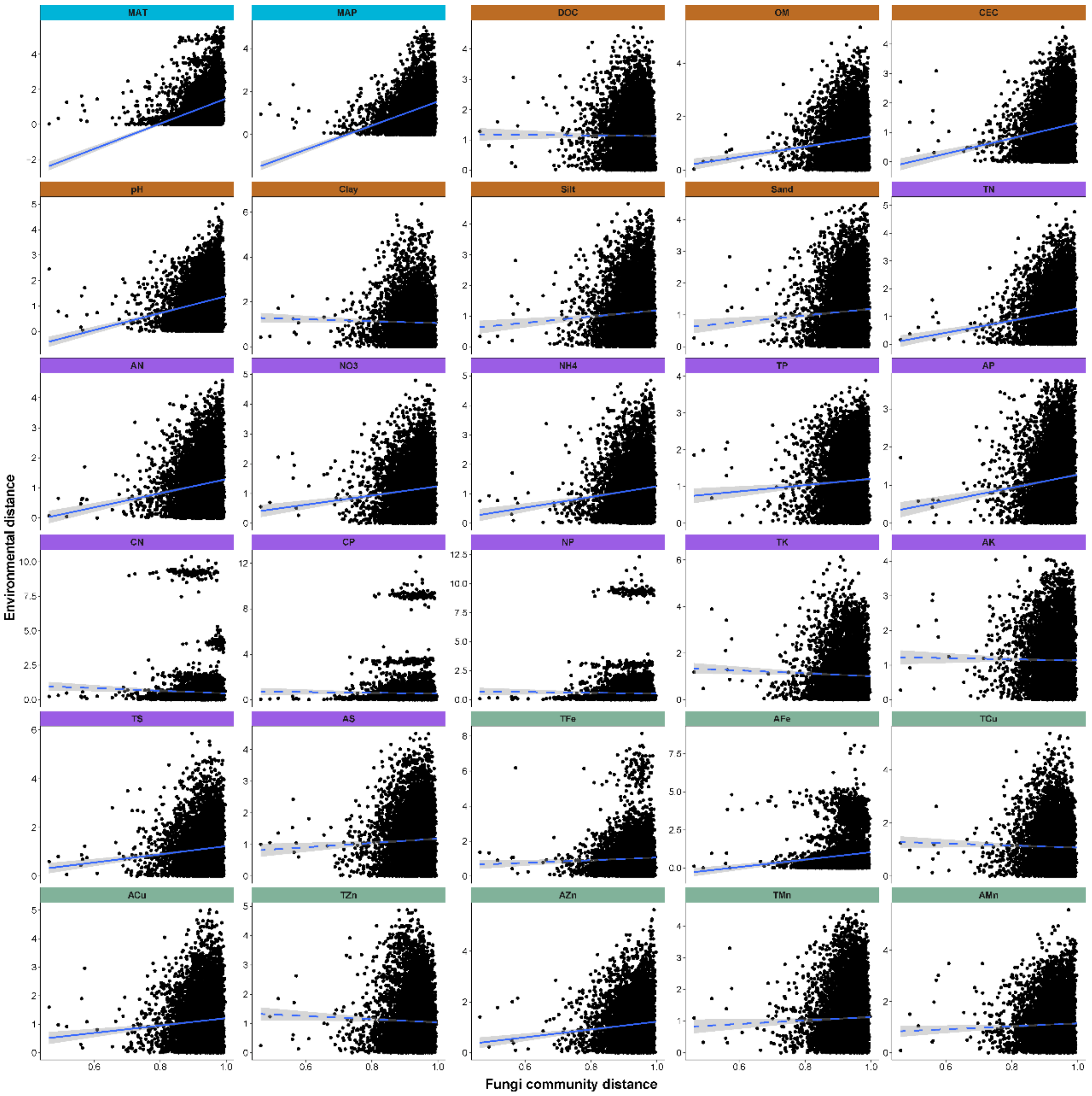

Supplementary Figure 7. Relationships between soil fungal communities and environmental distance in paddy soils. Dashed lines represent non-significant relationships.

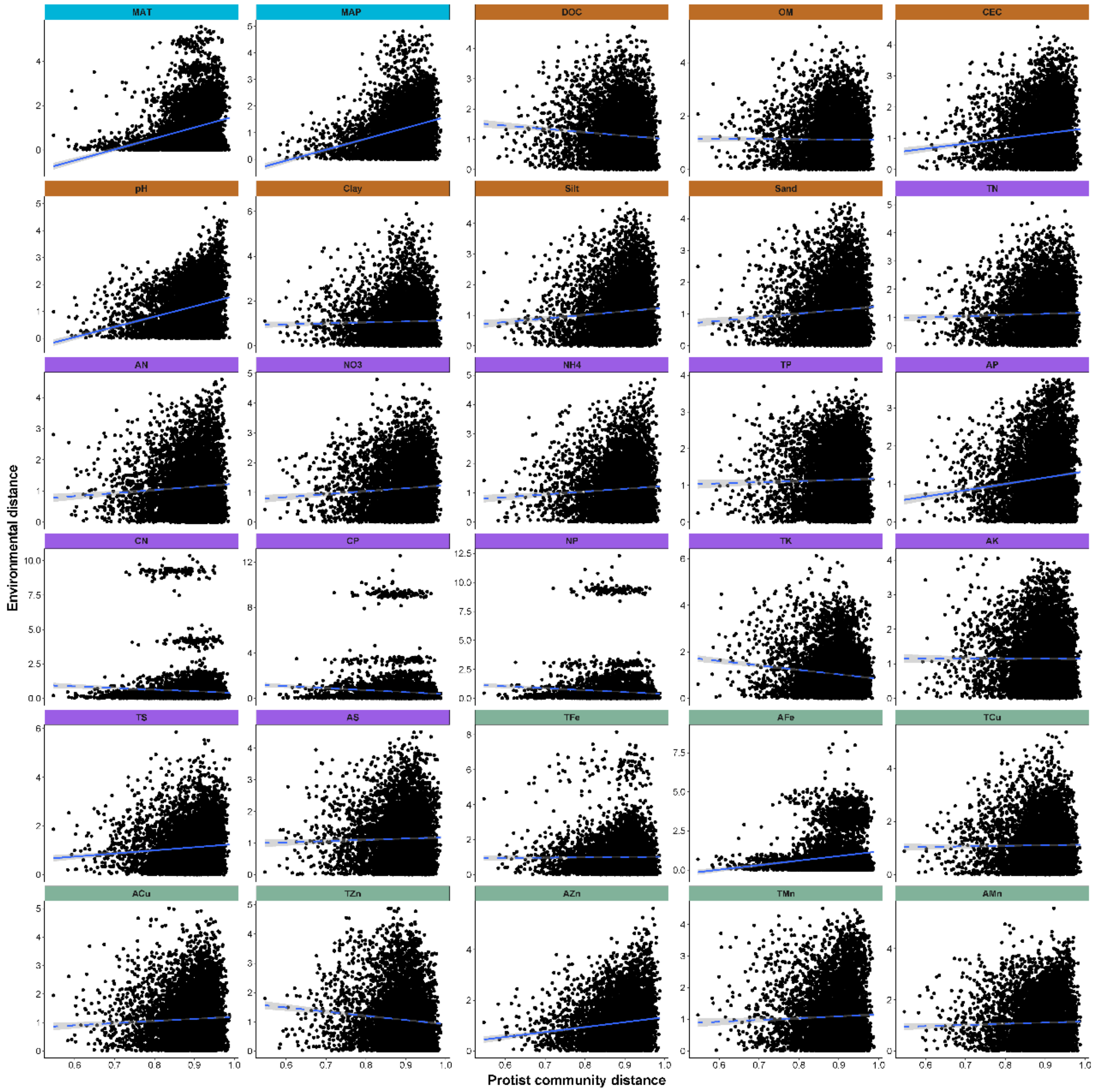

Supplementary Figure 8. Relationships between soil protistan communities and environmental distance in paddy soils. Dashed lines represent non-significant relationships.

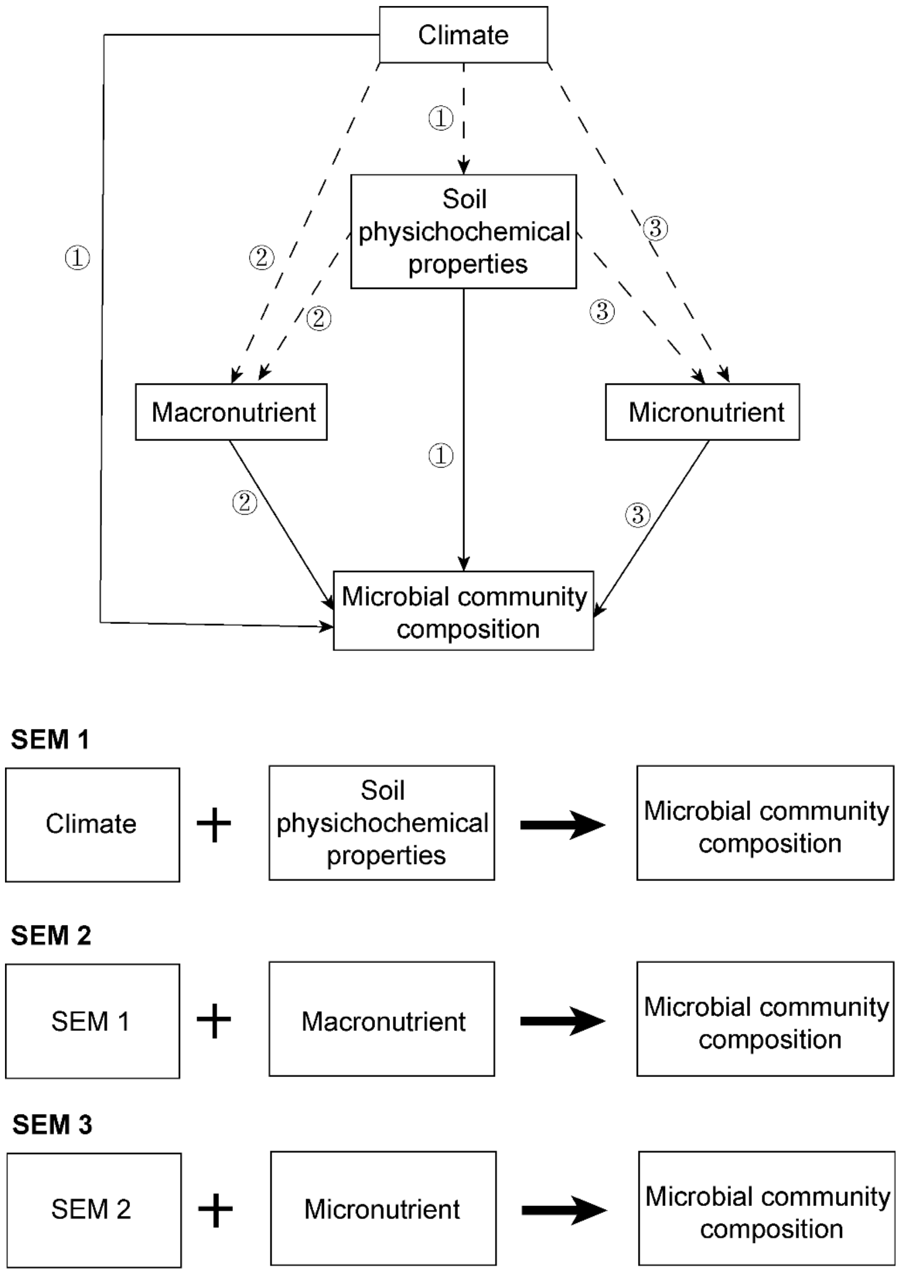

Supplementary Figure 9. The scheme depicting the methodological approach to examine the predictors of the structure of microbial communities. The theoretical direct (full lines) and indirect paths (dotted lines) from different variables to microbial communities that were tested in SEMs. The numbers indicate the order paths in which the influence of different variables was examined (1, climate and soil physicochemical properties; 2, including macronutrient; 3, including micronutrient).

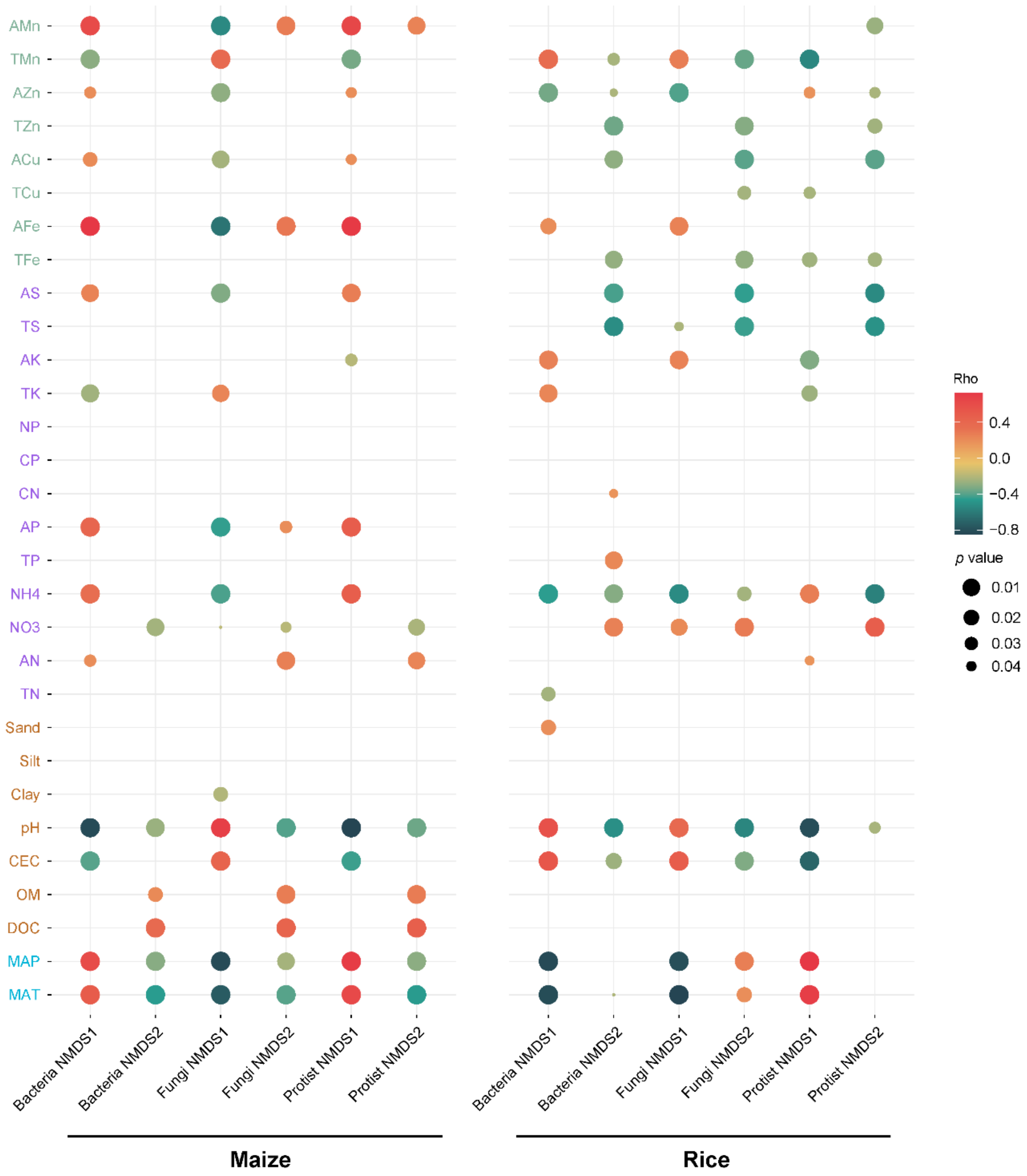

Supplementary Figure 10. Spearman correlation between environmental variables and the structure of bacterial, fungal and protistan communities (NMDS axes). Points represent correlation coefficients (Rho) and their significance ($p < 0.05$)

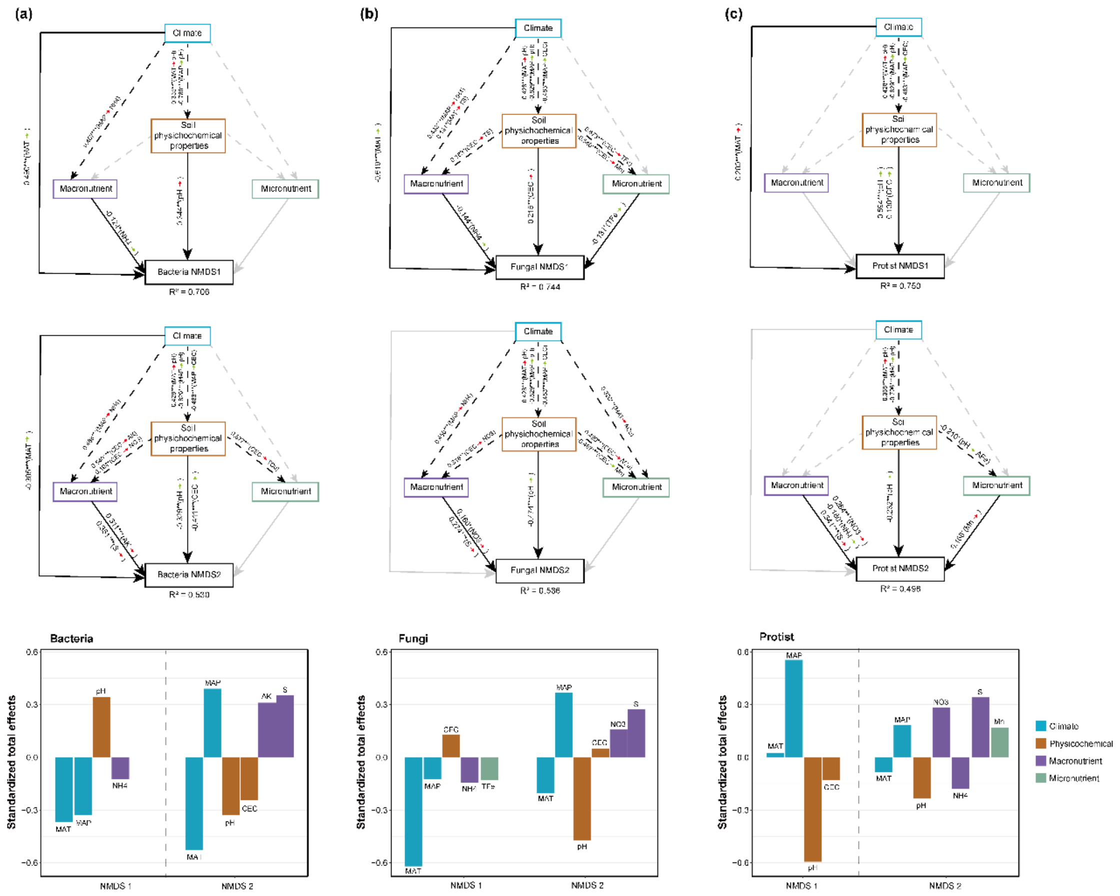

Supplementary Figure 11. Structural equation models identifying the direct (full lines) and indirect (dotted lines) influence of different predictors on the structure of bacterial (a), fungal (b) and protistan (c) communities in paddy soils. Black lines indicate significant and numbers adjacent to lines were indicative of the effect size of the relationship. We grouped the climatic and edaphic properties into the same box in the model for graphical simplicity, which did not represent latent variables. R^2 denotes the proportion of variance explained. Red arrows represented positive paths, and green arrows represented negative paths. Significance levels were as follows: *, $p < 0.05$, **, $p < 0.01$, and ***, $p < 0.001$. The total standardized effects on SEM on the structure of microbial communities were calculated as sum of the direct and indirect effects. Information about our a priori model was provided in Figureure S8.

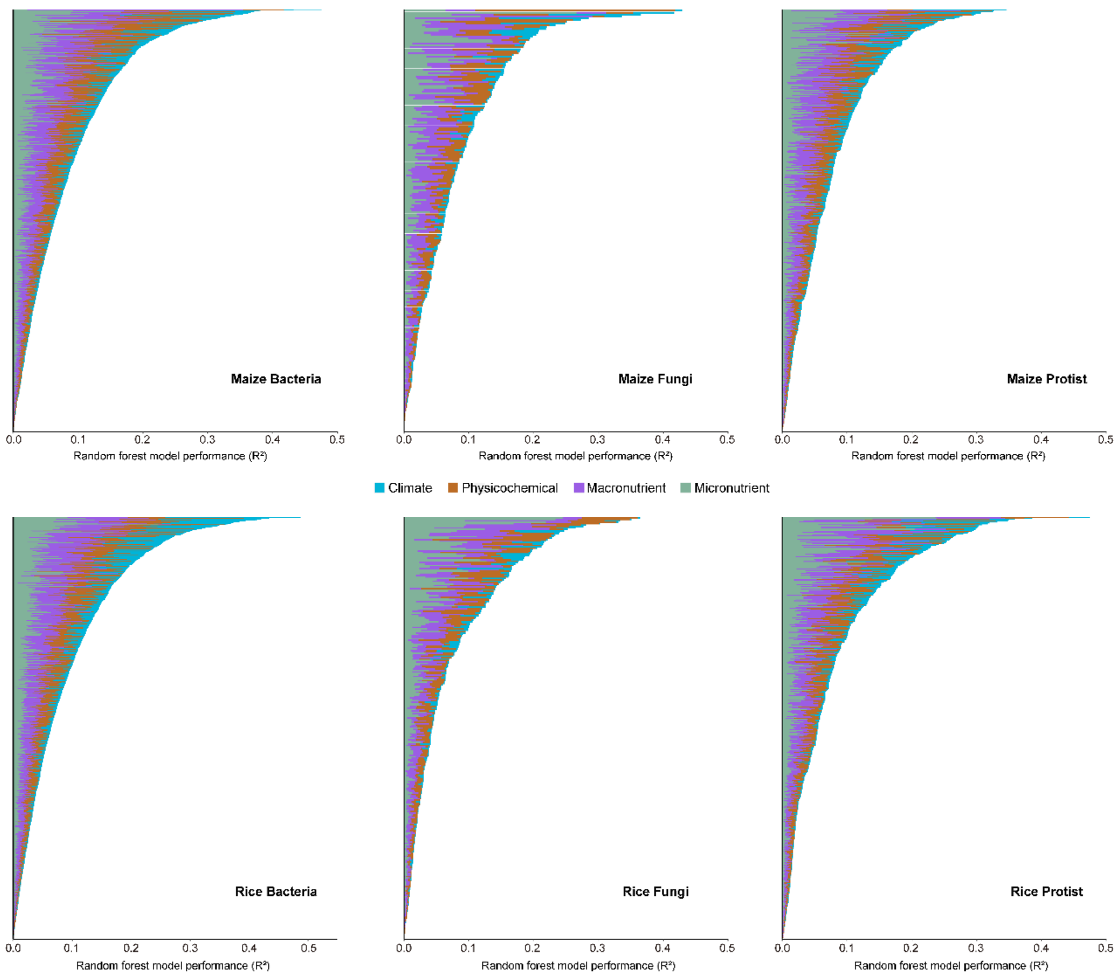

Supplementary Figure 12. Environmental variables explaining the structure of bacterial, fungal and protistan communities. Random forest model performance for microbial phylotypes (ASVs) where out-of-bag $R^2 > 0$.

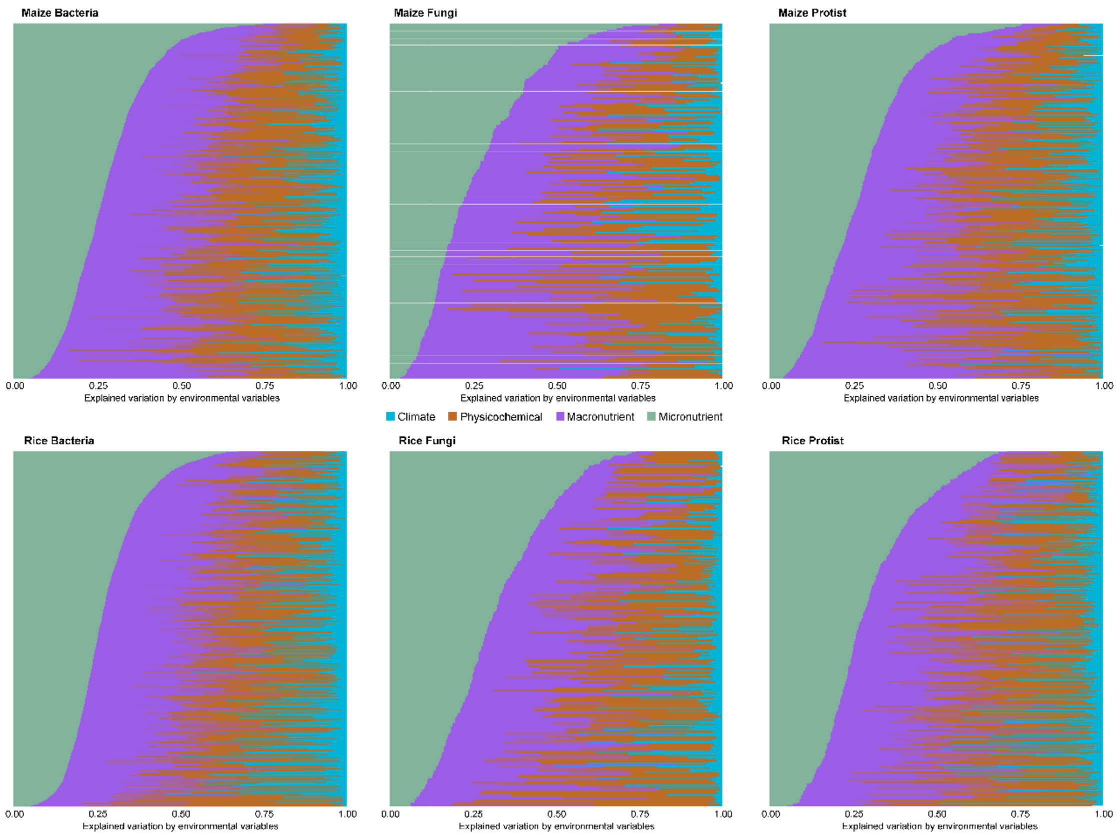

Supplementary Figure 13. Environmental variables explaining the structure of bacterial, fungal and protistan communities. Contribution of climate, soil physicochemical properties, macronutrients and micronutrients to the variation explained by the complete random forest model for each microbial phylotypes (ASVs) where out-of-bag $R^2 > 0$.

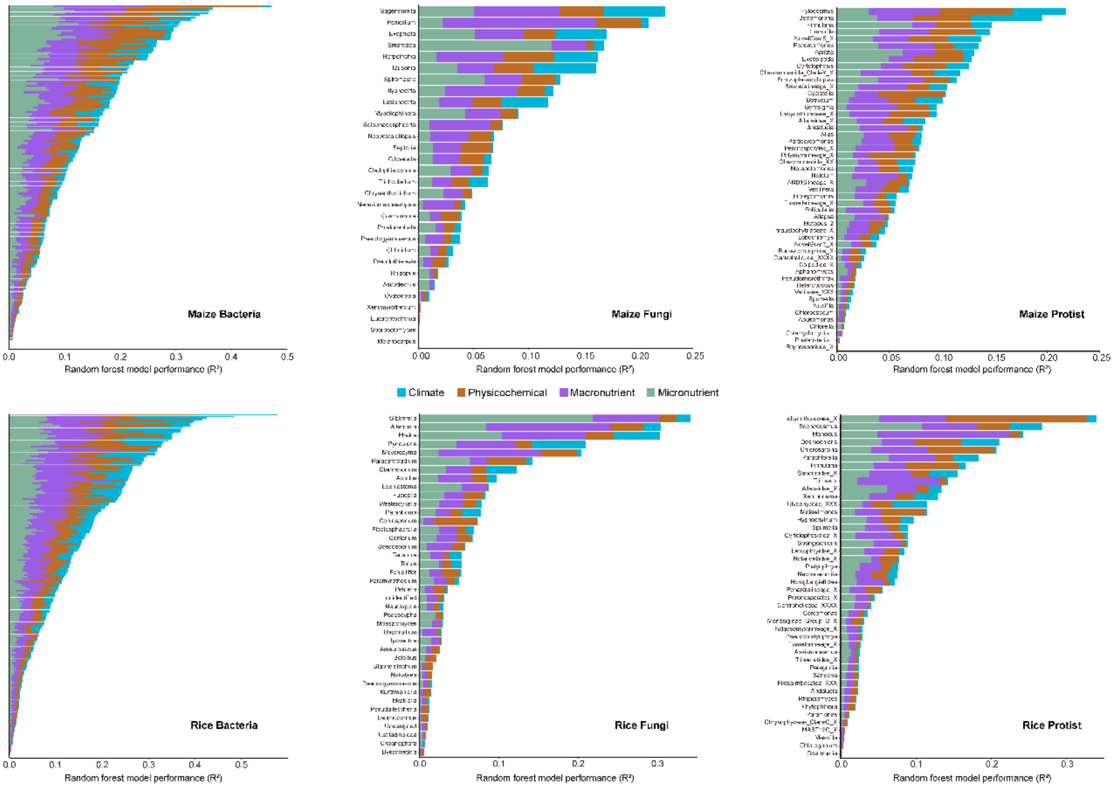

Supplementary Figure 14. Environmental variables explaining the structure of bacterial, fungal and protistan communities. Random forest model performance for microbial genera where out-of-bag $R^2 > 0$.

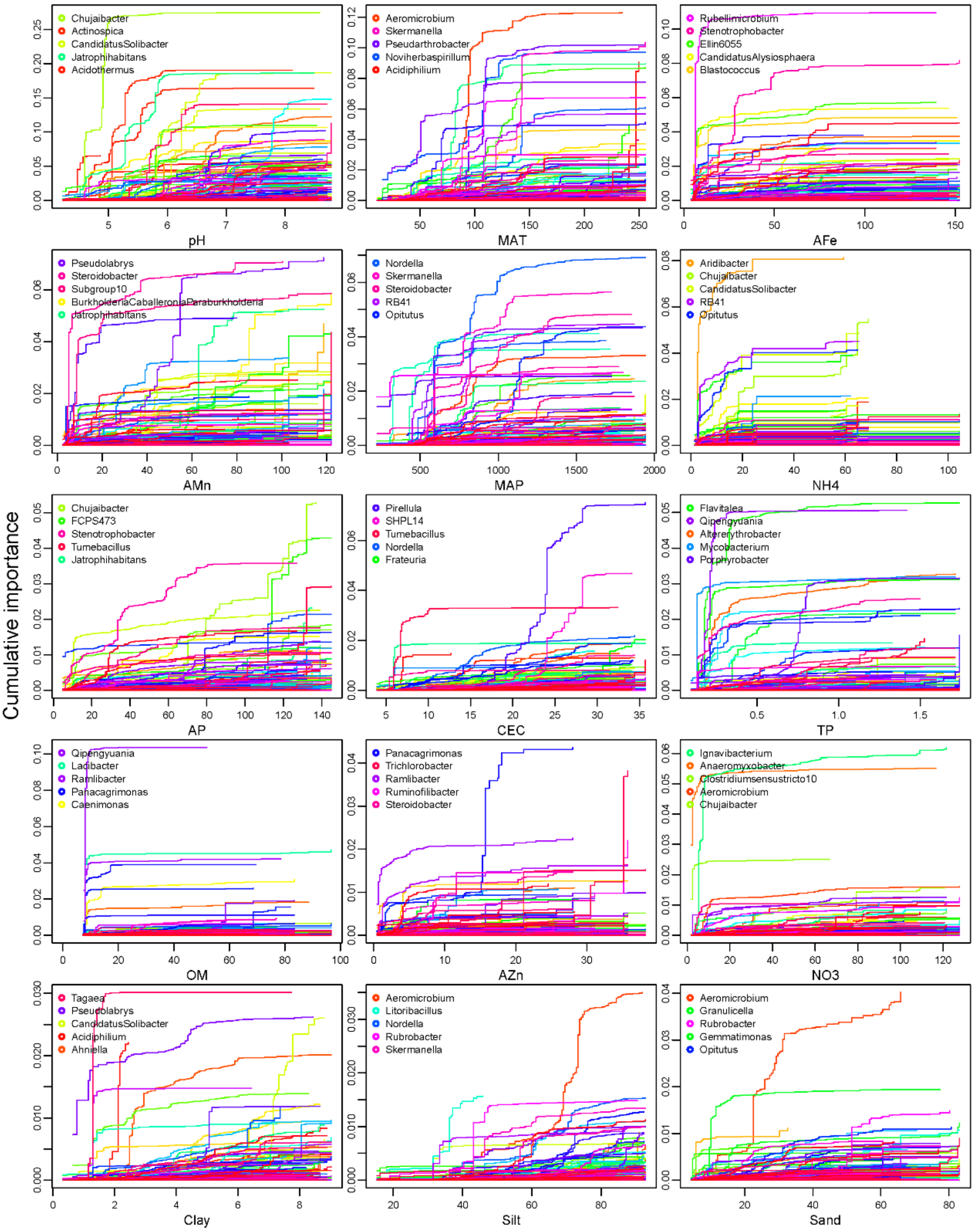

Supplementary Figure 15. Compositional turnover functions for these most important 15 predictors for bacterial genera in maize soils. Each genus shows cumulative importance distributions of splits improvement scaled by R^2 weighted importance. The top 5 most responsive species for each predictor were showed.

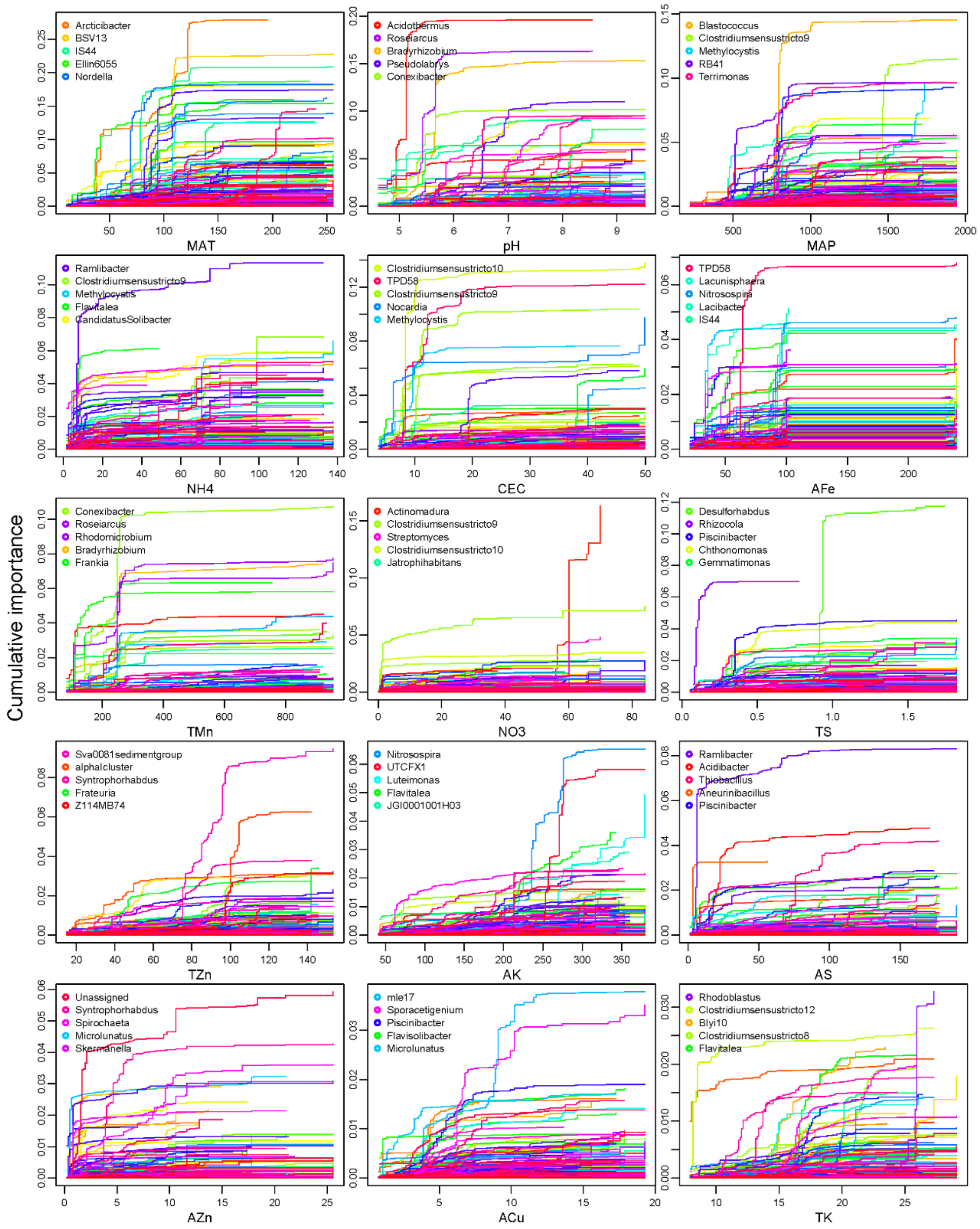

Supplementary Figure 16. Compositional turnover functions for these most important 15 predictors for bacterial genera in paddy soils. Each genus shows cumulative importance distributions of splits improvement scaled by R^2 weighted importance. The top 5 most responsive species for each predictor were showed.

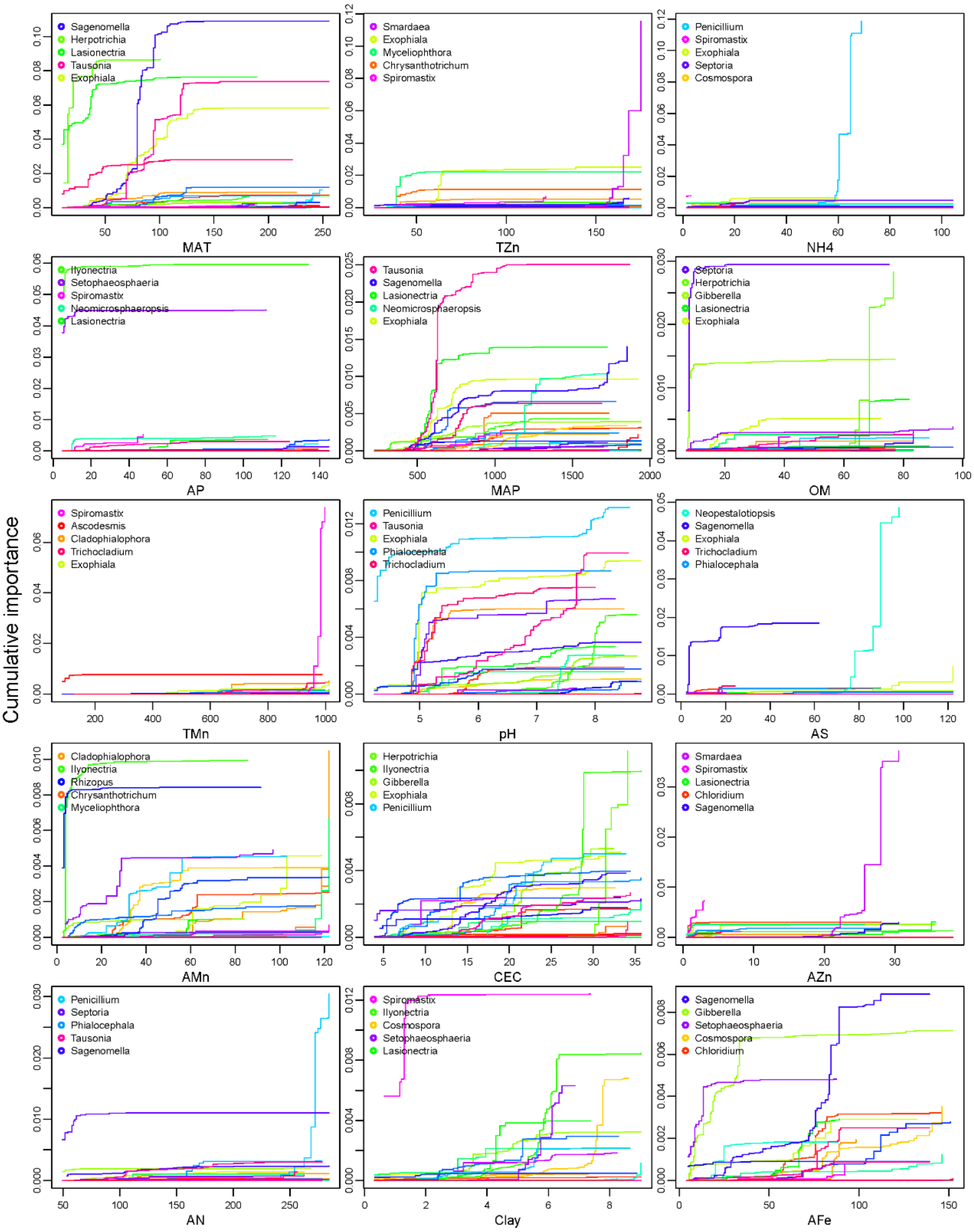

Supplementary Figure 17. Compositional turnover functions for these most important 15 predictors for fungal genera in maize soils. Each genus shows cumulative importance distributions of splits improvement scaled by R^2 weighted importance. The top 5 most responsive species for each predictor were showed.

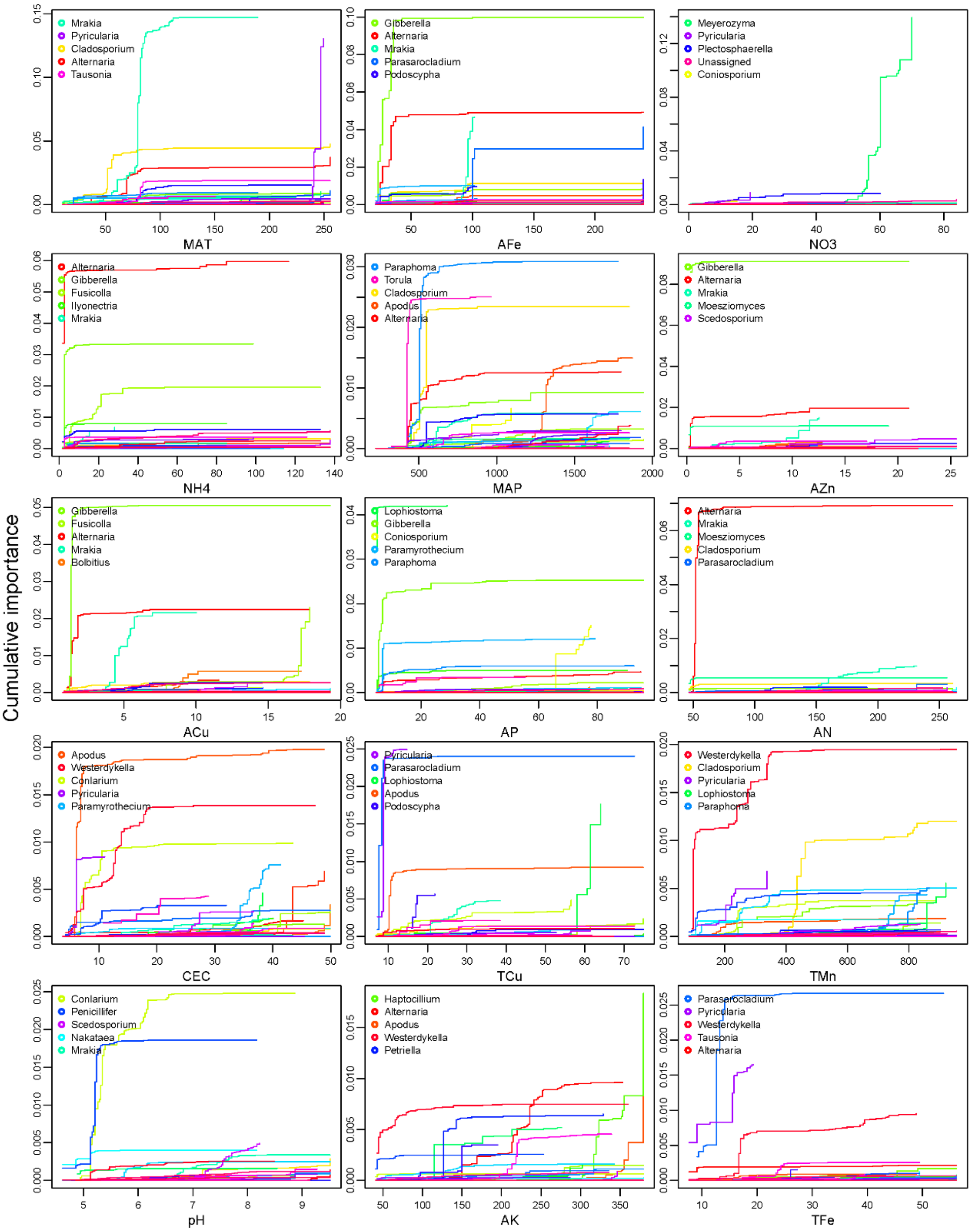

Supplementary Figure 18. Compositional turnover functions for these most important 15 predictors for fungal genera in paddy soils. Each genus shows cumulative importance distributions of splits improvement scaled by R^2 weighted importance. The top 5 most responsive species for each predictor were showed.

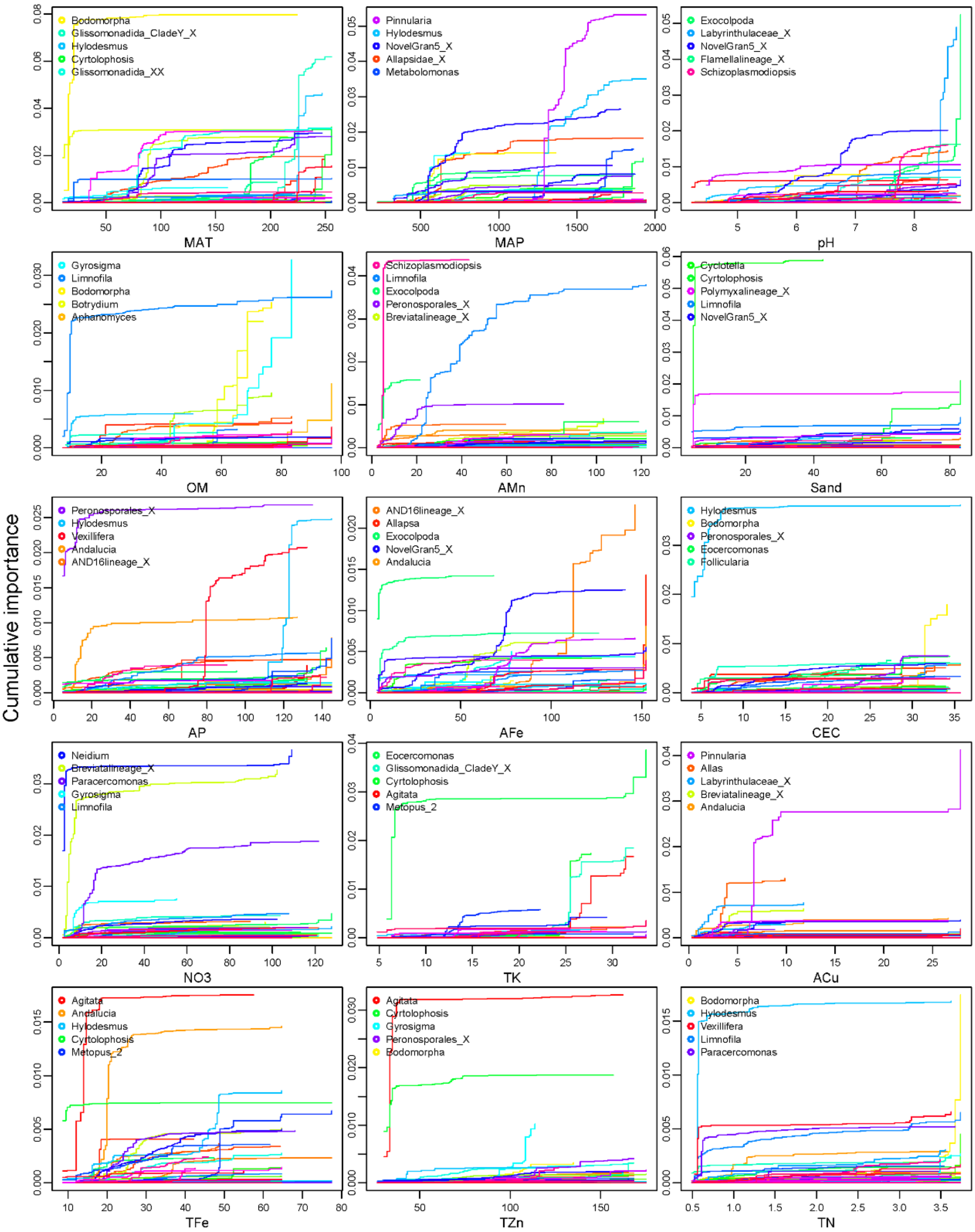

Supplementary Figure 19. Compositional turnover functions for these most important 15 predictors for protistan genera in maize soils. Each genus shows cumulative importance distributions of splits improvement scaled by R^2 weighted importance. The top 5 most responsive species for each predictor were showed.

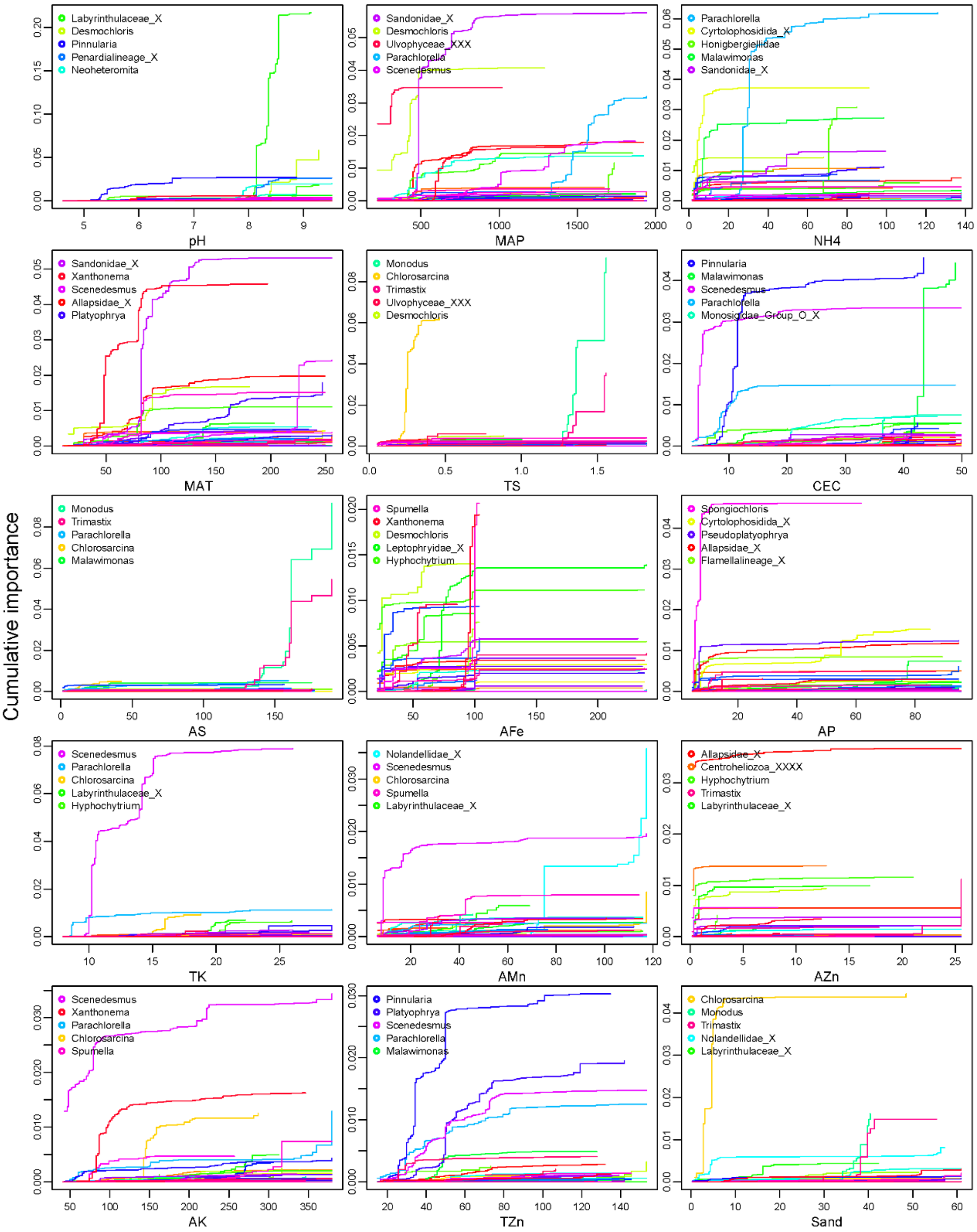

Supplementary Figure 20. Compositional turnover functions for these most important 15 predictors for protistan genera in paddy soils. Each genus shows cumulative importance distributions of splits improvement scaled by R^2 weighted importance. The top 5 most responsive species for each predictor were showed.

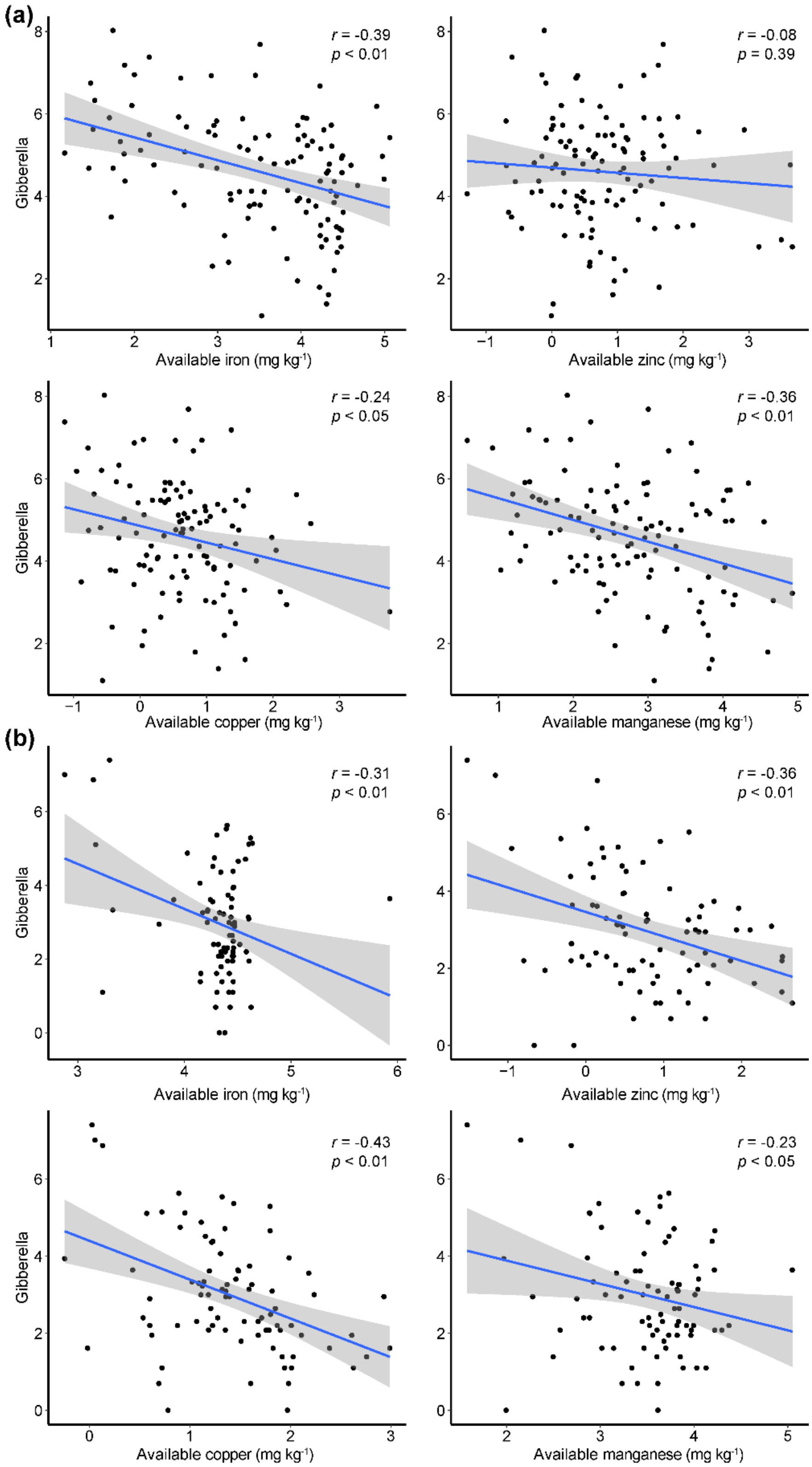

Supplementary Figure 21. The Pearson relationship between the available micronutrient composite and the relative abundance of Gibberella (log) in maize (a) and paddy (b) soils.

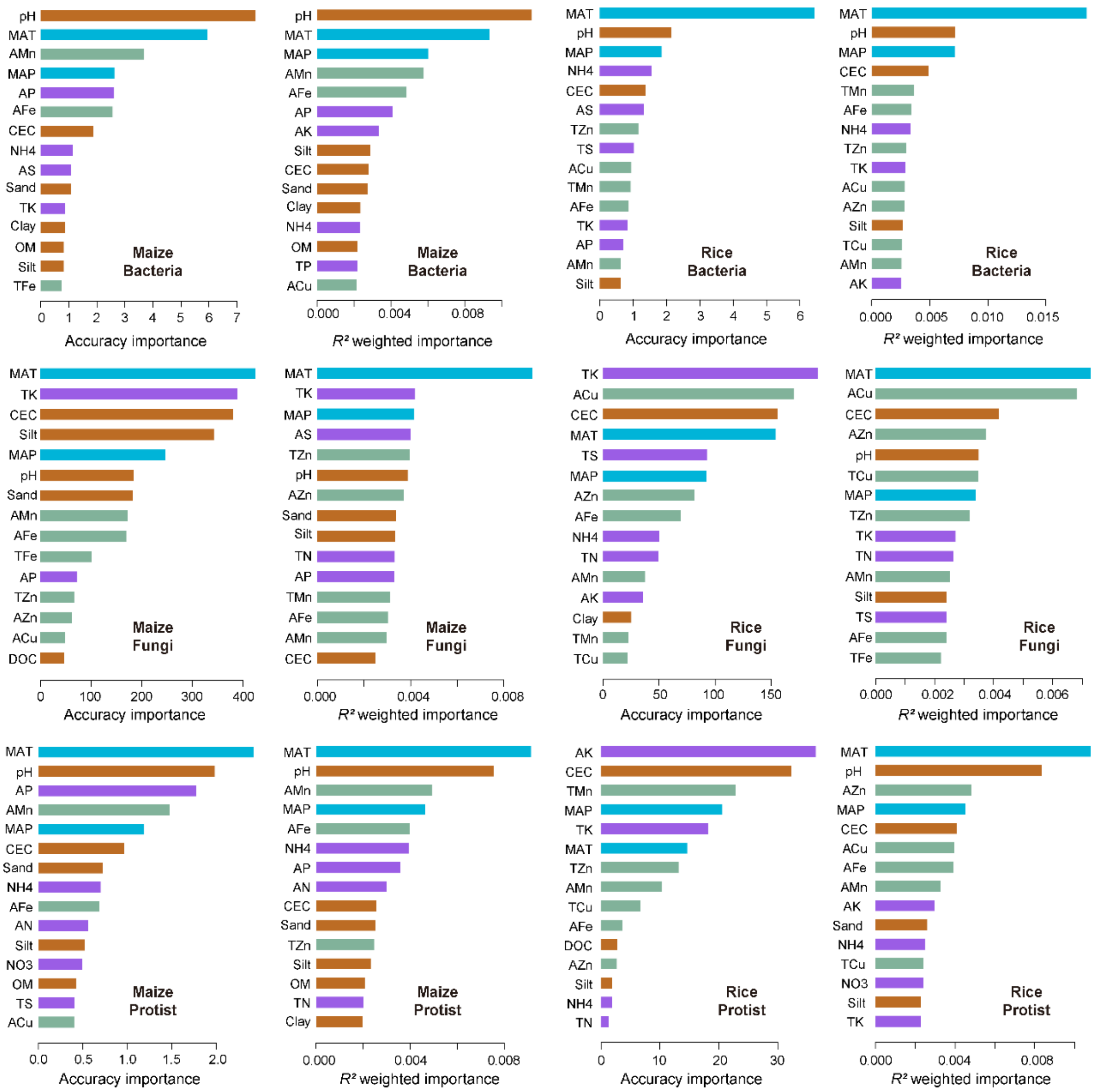

Supplementary Figure 22. Environmental variables explaining the structure of bacterial, fungal and protistan communities. Accuracy importance of individual environmental variables across models for microbial phylotypes showing raw variable importance and variable importance weighted by out-of-bag R^2 for each phylotypes.

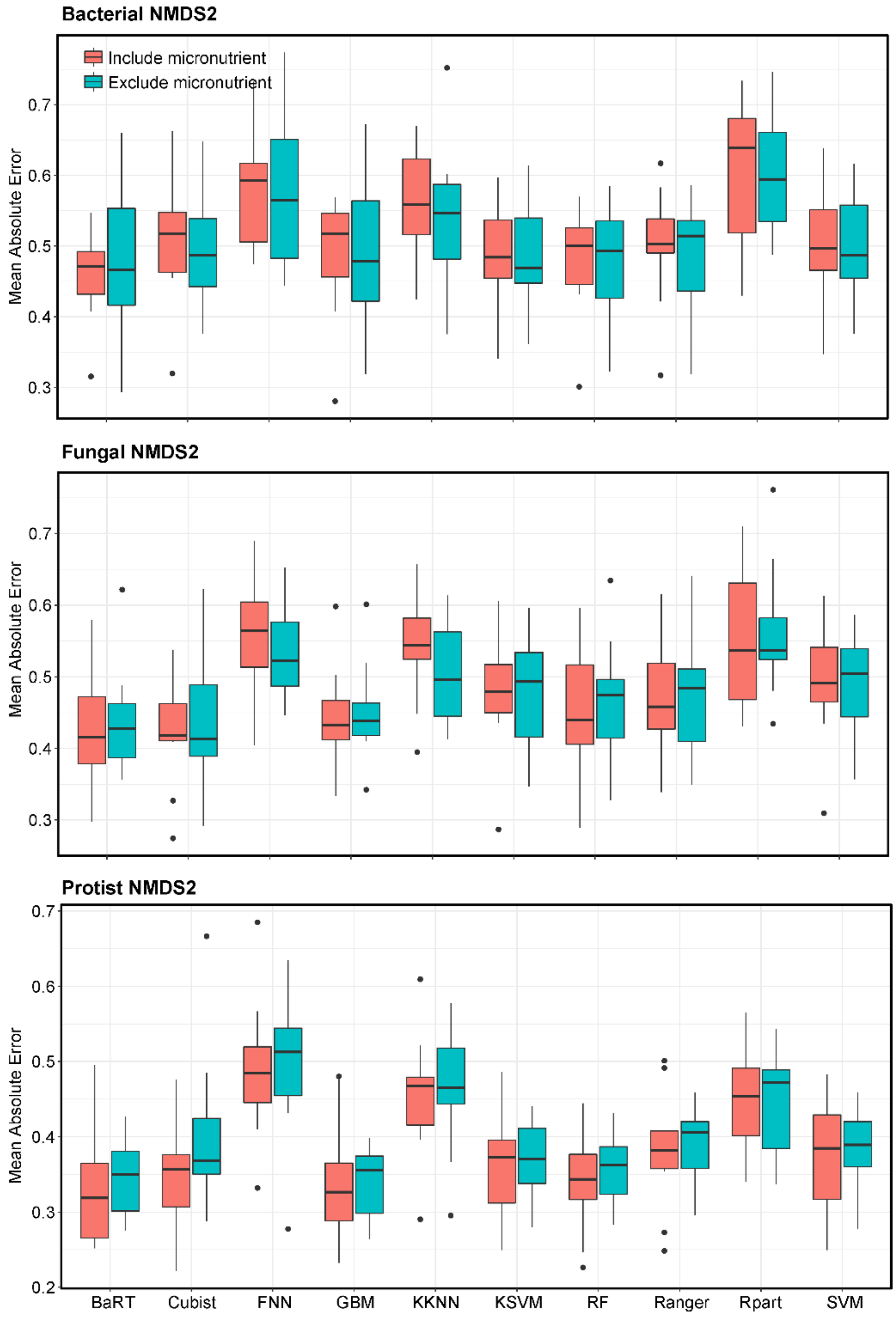

Supplementary Figure 23. Comparison of prediction errors of different machine learning algorithms for the structure of bacterial (a), fungal (c) and protistan (e) communities. Machine learning algorithms include Bagged Regression Tree (BaRT), Cubist, Fast Nearest Neighbor (FNN), Gradient Boosting Machines (GBM), Weighted k-Nearest Neighbor (KKNN), Kernel Support Vector Machine (KSVM), Random Forest (RF), Ranger, Rpart and Support Vector Machine (SVM).

Supplementary Table 1. Explained variance of bacterial, fungal and protistan communities in maize and paddy soils by environmental parameters using partial mantel test analysis and multiple regression on distance matrices. Four explanatory matrices: climate, soil physicochemical properties, macronutrients and micronutrients. ***, $p < 0.001$; **, $p < 0.01$; *, $p < 0.05$.

			Maize		Paddy	
	Taxa	Explanatory	*R*	*P*	*R*	*P*
Partial mantel	bacteria	climate	0.109	*	0.391	***
		soil	0.164	***	0.120	**
		macronutrients	0.033		0.063	
		micronutrients	0.117	**	0.044	
	fungi	climate	0.203	***	0.443	***
		soil	0.026	***	0.087	*
		macronutrients	0.045	*	0.128	**
		micronutrients	0.184	***	-0.030	
	protist	climate	0.258	***	0.307	***
		soil	0.087	*	0.114	***
		macronutrients	0.156	**	0.002	
		micronutrients	0.171	***	0.042	
	Taxa	Explanatory	R^2	*P*	R^2	*P*
MRM	bacteria	climate	0.106	*	0.385	***
		soil	0.181	***	0.119	**
		macronutrients	0.034		0.059	
		micronutrients	0.126	*	0.043	
	fungi	climate	0.200	***	0.437	***
		soil	0.029		0.083	*
		macronutrients	0.046		0.119	**
		micronutrients	0.200	**	-0.028	
	protist	climate	0.247	***	0.305	***
		soil	0.090	*	0.117	*
		macronutrients	0.153	**	0.002	
		micronutrients	0.178	***	0.043	

Supplementary Table 2. Mantel tests for the relationship between each environmental predictor and the structure of bacterial, fungal, and protistan communities in maize soils.

		Bacteria		Fungi		Protist	
Environment	Each predictor	*R*	*P*	*R*	*P*	*R*	*P*
Climate	MAT	0.086	0.059	0.103	0.033	0.165	0.002
	MAP	0.180	0.000	0.281	0.000	0.336	0.000
Physiochemical	pH	0.488	0.000	0.268	0.000	0.375	0.000
	CEC	0.120	0.003	0.091	0.015	0.130	0.001
	OM	0.041	0.173	0.063	0.079	0.112	0.007
	DOC	0.049	0.124	-0.008	0.560	0.050	0.114
	Clay	0.095	0.023	0.027	0.284	0.026	0.287
	Silt	0.007	0.432	-0.007	0.528	0.008	0.430
	Sand	0.020	0.331	-0.002	0.504	0.015	0.365
Macronutrient	TN	0.046	0.130	0.085	0.025	0.066	0.057
	AN	0.073	0.044	0.132	0.001	0.147	0.001
	NO3	0.056	0.090	0.089	0.018	0.091	0.014
	NH4	0.121	0.015	0.062	0.126	0.039	0.214
	TP	-0.042	0.807	-0.034	0.755	-0.002	0.510
	AP	0.124	0.000	0.090	0.006	0.216	0.000
	CN	0.072	0.128	0.020	0.367	0.029	0.301
	CP	0.030	0.297	0.050	0.214	0.077	0.111
	NP	0.025	0.326	0.061	0.166	0.092	0.076
	TK	0.073	0.090	0.046	0.207	0.172	0.003
	AK	-0.013	0.607	0.018	0.344	0.097	0.018
	TS	0.034	0.210	0.028	0.255	-0.015	0.626
	AS	0.030	0.243	0.068	0.075	0.078	0.049
Micronutrient	TFe	0.065	0.115	0.159	0.003	0.141	0.008
	AFe	0.258	0.000	0.168	0.000	0.269	0.000
	TZn	0.091	0.016	0.090	0.023	0.072	0.054
	AZn	0.000	0.496	0.038	0.221	0.050	0.150
	TCu	0.101	0.020	0.105	0.019	0.089	0.039
	ACu	0.063	0.091	0.086	0.039	0.104	0.017
	TMn	0.120	0.009	0.120	0.009	0.190	0.001
	AMn	0.197	0.000	0.118	0.002	0.189	0.000

Supplementary Table 3. Mantel tests for the relationship between each environmental predictor and the structure of bacterial, fungal, and protistan communities in paddy soils.

		Bacteria		Fungi		Protist	
Environment	Each predictor	R	P	R	P	R	P
Climate	MAT	0.372	0.000	0.324	0.000	0.272	0.000
	MAP	0.301	0.000	0.309	0.000	0.276	0.000
Physiochemical	pH	0.261	0.000	0.188	0.000	0.262	0.000
	CEC	0.177	0.000	0.146	0.000	0.109	0.004
	OM	0.069	0.062	0.106	0.003	-0.006	0.550
	DOC	-0.039	0.817	-0.006	0.561	-0.075	0.960
	Clay	-0.041	0.793	-0.022	0.692	0.027	0.301
	Silt	0.122	0.003	0.055	0.075	0.077	0.055
	Sand	0.109	0.008	0.056	0.077	0.075	0.058
Macronutrient	TN	0.074	0.044	0.121	0.001	0.027	0.273
	AN	0.101	0.013	0.127	0.001	0.066	0.084
	NO3	0.071	0.039	0.085	0.008	0.067	0.056
	NH4	0.121	0.005	0.099	0.007	0.064	0.089
	TP	0.036	0.092	0.049	0.025	0.022	0.222
	AP	0.166	0.000	0.098	0.002	0.115	0.002
	CN	-0.076	0.903	-0.034	0.722	-0.052	0.781
	CP	-0.085	0.937	-0.012	0.560	-0.079	0.885
	NP	-0.078	0.912	-0.012	0.558	-0.074	0.866
	TK	0.006	0.448	-0.031	0.751	-0.117	0.987
	AK	0.035	0.179	-0.010	0.602	-0.001	0.494
	TS	0.119	0.008	0.090	0.015	0.084	0.045
	AS	0.022	0.288	0.038	0.140	0.027	0.268
Micronutrient	TFe	0.116	0.023	0.035	0.220	0.010	0.424
	AFe	0.096	0.051	0.102	0.024	0.149	0.009
	TZn	0.020	0.331	-0.027	0.732	-0.089	0.958
	AZn	0.106	0.013	0.084	0.020	0.128	0.006
	TCu	0.004	0.462	-0.021	0.691	0.014	0.385
	ACu	0.087	0.032	0.070	0.039	0.050	0.154
	TMn	0.109	0.010	0.029	0.237	0.037	0.222
	AMn	0.063	0.086	0.030	0.216	0.030	0.253

Supplementary Table 4. Comparison of prediction errors of different machine learning algorithms for the structure of bacterial communities. BaRT, Bagged Regression Tree; FNN, Fast Nearest Neighbor; GBM, Gradient Boosting Machines; KKNN, Weighted k-Nearest Neighbor; KSVM, Kernel Support Vector Machine; RF, Random Forest; SVM, Support Vector Machine. MAE, mean absolute error; MSE, mean squared error; RMSE, root mean squared error.

	Include micronutrient						Exclude micronutrient					
	NMDS1			NMDS2			NMDS1			NMDS2		
	MAE	MSE	RMSE	MAE	MSE	RMSE	MAE	MSE	RMSE	MAE	MSE	RMSE
BaRT	0.445	0.361	0.593	0.459	0.346	0.581	0.512	0.460	0.674	0.480	0.374	0.599
Cubist	0.465	0.380	0.610	0.506	0.424	0.642	0.541	0.506	0.705	0.495	0.395	0.619
FNN	0.541	0.492	0.690	0.582	0.545	0.730	0.587	0.568	0.747	0.576	0.543	0.727
RF	0.446	0.353	0.584	0.479	0.390	0.615	0.518	0.459	0.674	0.470	0.376	0.603
GBM	0.470	0.397	0.623	0.487	0.385	0.609	0.518	0.467	0.680	0.487	0.394	0.616
KKNN	0.520	0.480	0.683	0.563	0.519	0.713	0.586	0.549	0.734	0.539	0.484	0.685
KSVM	0.465	0.388	0.616	0.487	0.388	0.615	0.528	0.482	0.688	0.483	0.385	0.611
SVM	0.473	0.400	0.625	0.500	0.412	0.633	0.536	0.489	0.693	0.496	0.406	0.628
Rpart	0.544	0.520	0.705	0.605	0.612	0.773	0.652	0.697	0.833	0.602	0.567	0.746
Ranger	0.466	0.382	0.606	0.499	0.418	0.637	0.531	0.476	0.686	0.484	0.391	0.615

Supplementary Table 5. Comparison of prediction errors of different machine learning algorithms for the structure of fungal communities.

	Include micronutrient						Exclude micronutrient					
	NMDS1			NMDS2			NMDS1			NMDS2		
	MAE	MSE	RMSE	MAE	MSE	RMSE	MAE	MSE	RMSE	MAE	MSE	RMSE
BaRT	0.409	0.284	0.525	0.425	0.306	0.545	0.484	0.419	0.635	0.439	0.313	0.551
Cubist	0.454	0.331	0.568	0.423	0.305	0.543	0.515	0.495	0.692	0.437	0.315	0.550
FNN	0.461	0.365	0.594	0.558	0.493	0.695	0.549	0.492	0.689	0.537	0.462	0.676
RF	0.430	0.293	0.533	0.452	0.346	0.574	0.512	0.441	0.658	0.465	0.352	0.583
GBM	0.430	0.303	0.542	0.438	0.317	0.555	0.473	0.405	0.626	0.451	0.327	0.566
KKNN	0.448	0.350	0.583	0.542	0.477	0.684	0.534	0.464	0.671	0.501	0.419	0.643
KSVM	0.438	0.323	0.558	0.474	0.379	0.606	0.522	0.463	0.672	0.477	0.370	0.600
SVM	0.447	0.337	0.570	0.490	0.404	0.626	0.532	0.478	0.683	0.488	0.388	0.616
Rpart	0.514	0.496	0.692	0.552	0.503	0.697	0.554	0.593	0.761	0.561	0.517	0.712
Ranger	0.451	0.321	0.559	0.469	0.365	0.592	0.538	0.468	0.678	0.473	0.364	0.593

Supplementary Table 6. Comparison of prediction errors of different machine learning algorithms for the structure of protistan communities.

	Include micronutrient						Exclude micronutrient					
	NMDS1			NMDS2			NMDS1			NMDS2		
	MAE	MSE	RMSE	MAE	MSE	RMSE	MAE	MSE	RMSE	MAE	MSE	RMSE
BaRT	0.450	0.316	0.559	0.334	0.219	0.452	0.494	0.398	0.622	0.345	0.234	0.477
Cubist	0.442	0.312	0.554	0.343	0.235	0.474	0.515	0.487	0.672	0.405	0.443	0.597
FNN	0.537	0.480	0.691	0.489	0.456	0.655	0.573	0.544	0.723	0.495	0.458	0.666
RF	0.464	0.334	0.575	0.343	0.239	0.476	0.523	0.428	0.646	0.357	0.250	0.491
GBM	0.482	0.356	0.594	0.339	0.222	0.456	0.505	0.409	0.631	0.338	0.227	0.469
KKNN	0.509	0.426	0.650	0.457	0.412	0.624	0.571	0.528	0.718	0.462	0.411	0.633
KSVM	0.466	0.353	0.591	0.364	0.266	0.504	0.528	0.457	0.670	0.365	0.269	0.510
SVM	0.484	0.377	0.610	0.373	0.284	0.519	0.545	0.478	0.685	0.381	0.287	0.527
Rpart	0.485	0.403	0.623	0.451	0.382	0.606	0.580	0.582	0.748	0.447	0.357	0.592
Ranger	0.485	0.354	0.593	0.381	0.278	0.514	0.543	0.449	0.663	0.388	0.281	0.522
