# Supplementary material for: The neglected role of micronutrients in predicting soil microbial structure
Source: NPJ Biofilms Microbiomes. 2022 Dec 27;8:103. doi: 10.1038/s41522-022-00363-3 (PMC9794713; doi:10.1038/s41522-022-00363-3)
Supplement: Supplementary file 2 — Reporting Summary [file 41522_2022_363_MOESM2_ESM.pdf]

nature portfolio

Corresponding author(s): Dr. Gehong Wei, Dr. Shuo Jiao

Last updated by author(s): Oct 17, 2022

Reporting Summary

Nature Portfolio wishes to improve the reproducibility of the work that we publish. This form provides structure for consistency and transparency in reporting. For further information on Nature Portfolio policies, see our Editorial Policies and the Editorial Policy Checklist.

Statistics

For all statistical analyses, confirm that the following items are present in the figure legend, table legend, main text, or Methods section.

n/a	Confirmed	
☐	☒	The exact sample size (*n*) for each experimental group/condition, given as a discrete number and unit of measurement
☐	☒	A statement on whether measurements were taken from distinct samples or whether the same sample was measured repeatedly
☐	☒	The statistical test(s) used AND whether they are one- or two-sided<br>*Only common tests should be described solely by name; describe more complex techniques in the Methods section.*
☐	☒	A description of all covariates tested
☐	☒	A description of any assumptions or corrections, such as tests of normality and adjustment for multiple comparisons
☐	☒	A full description of the statistical parameters including central tendency (e.g. means) or other basic estimates (e.g. regression coefficient) AND variation (e.g. standard deviation) or associated estimates of uncertainty (e.g. confidence intervals)
☐	☒	For null hypothesis testing, the test statistic (e.g. F, t, r) with confidence intervals, effect sizes, degrees of freedom and P value noted<br>*Give P values as exact values whenever suitable.*
☒	☐	For Bayesian analysis, information on the choice of priors and Markov chain Monte Carlo settings
☒	☐	For hierarchical and complex designs, identification of the appropriate level for tests and full reporting of outcomes
☐	☒	Estimates of effect sizes (e.g. Cohen's *d*, Pearson's *r*), indicating how they were calculated

Our web collection on statistics for biologists contains articles on many of the points above.

Software and code

Policy information about availability of computer code

Data collection	We combined the 114 parallel agricultural fields (maize and rice) survey of soil bacteria, fungi and protist with environmental data in eastern China.
Data analysis	All statistical analyses were performed using R 4.1.0.

For manuscripts utilizing custom algorithms or software that are central to the research but not yet described in published literature, software must be made available to editors and reviewers. We strongly encourage code deposition in a community repository (e.g. GitHub). See the Nature Portfolio guidelines for submitting code & software for further information.

Data

Policy information about availability of data

All manuscripts must include a data availability statement. This statement should provide the following information, where applicable:

- Accession codes, unique identifiers, or web links for publicly available datasets
- A description of any restrictions on data availability
- For clinical datasets or third party data, please ensure that the statement adheres to our policy

Environmental data are archived in https://doi.org/10.6084/m9.figshare.19765978.v1. Sequencing data are accessible on GenBank via BioProject accession PRJNA544819 at https://www.ncbi.nlm.nih.gov/bioproject/?term=PRJNA544819.

Sequencing data are accessible on GenBank via BioProject accession PRJNA544819 at https://www.ncbi.nlm.nih.gov/bioproject/?term=PRJNA544819.

Human research participants

Policy information about studies involving human research participants and Sex and Gender in Research.

Reporting on sex and gender	n/a
Population characteristics	n/a
Recruitment	n/a
Ethics oversight	n/a

Note that full information on the approval of the study protocol must also be provided in the manuscript.

Field-specific reporting

Please select the one below that is the best fit for your research. If you are not sure, read the appropriate sections before making your selection.

☐ Life sciences ☐ Behavioural & social sciences ☒ Ecological, evolutionary & environmental sciences

For a reference copy of the document with all sections, see nature.com/documents/nr-reporting-summary-flat.pdf

Ecological, evolutionary & environmental sciences study design

All studies must disclose on these points even when the disclosure is negative.

Study description	Here, we used data from 228 agricultural fields to identify the importance of micronutrients (iron, zinc, copper and manganese) in shaping structure of soil microbial communities (bacteria, fungi and protist) along latitudinal gradient over 3400 km, across diverse edaphic conditions and climate gradients.
Research sample	A total of 228 soil samples were analyzed in this study.
Sampling strategy	Collected paddy soils are around maize soils (within 5 km) in each paired site (Supplementary Fig. 1). The soil samples were collected during the growing season (July–September 2017). In each site, a composite soil sample were composed of 15 soil cores taken at a depth of 0–15 cm from three plots (each 100 m2) and was separated into two portions.
Data collection	We used data from 114 parallel agricultural fields (maize and rice) in eastern China, covering diverse climate and edaphic gradients. This data contained a set of 30 environmental variables-climate (annual mean temperature and precipitation), soil properties (soil pH, cation exchange capacity, sand, silt, clay, dissolved and total organic matter), macronutrients (total and available nitrogen, phosphorus, potassium and sulfur, nitrate and ammonia nitrogen, C/N, C/P and N/P) and micronutrients (total and available iron, zinc, copper, manganese).
Timing and spatial scale	Sample collection of soils took place between July–September 2017.
Data exclusions	n/a
Reproducibility	Information about the sampled locations and methods used in this paper are included in our material and methods.
Randomization	n/a
Blinding	n/a

Did the study involve field work? ☐ Yes ☒ No

Reporting for specific materials, systems and methods

We require information from authors about some types of materials, experimental systems and methods used in many studies. Here, indicate whether each material, system or method listed is relevant to your study. If you are not sure if a list item applies to your research, read the appropriate section before selecting a response.

Materials & experimental systems

n/a	Involved in the study
☒	☐ Antibodies
☒	☐ Eukaryotic cell lines
☒	☐ Palaeontology and archaeology
☒	☐ Animals and other organisms
☒	☐ Clinical data
☒	☐ Dual use research of concern

Methods

n/a	Involved in the study
☒	☐ ChIP-seq
☒	☐ Flow cytometry
☒	☐ MRI-based neuroimaging
